# Supplementary material for: In Situ Detection of Adeno-associated Viral Vector Genomes with SABER-FISH
Source: Mol Ther Methods Clin Dev. 2020 Oct 10;19:376–86. doi: 10.1016/j.omtm.2020.10.003 (PMC7658570; doi:10.1016/j.omtm.2020.10.003)
Supplement: Document S1. Supplemental Materials and Methods, Figures S1–S6, and Tables S1–S6 [file mmc1.pdf]

OMTM, Volume *19*

Supplemental Information

In Situ Detection of Adeno-associated Viral Vector Genomes with SABER-FISH

Sean K. Wang, Sylvain W. Lapan, Christin M. Hong, Tyler B. Krause, and Constance L. Cepko

SUPPLEMENTAL METHODS

Cell sorting. Microglia from $CX3CR1^{GFP/+}$ mouse retinas were isolated using a BD FACSAria Fusion as previously described with minor modifications.[1] For each sample, four freshly dissected retinas were pooled and dissociated using cysteine-activated papain followed by gentle trituration with a micropipette. Samples were subsequently washed with FACS buffer (PBS containing 2% fetal bovine serum and 2mM ethylenediaminetetraacetic acid [EDTA]), passed through a 40 µm filter, and stained with 0.5 µg/mL of DAPI (Invitrogen) to exclude non-viable cells. Analysis of sorted populations was performed using FlowJo 10 (Tree Star).

qPCR. Total DNA including viral DNA was purified from sorted CX3CR1-positive microglia using a *Quick*-DNA Microprep Plus Kit (Zymo Research). Total RNA was purified from whole retinas (pre-sort) or sorted CX3CR1-positive microglia (post-sort) using an RNeasy Micro Kit (Qiagen). For RNA samples, cDNA was synthesized using the SuperScript III First-Strand Synthesis System (Invitrogen) with oligo(dT) primers. Total DNA or cDNA templates were then assayed by qPCR using the Power SYBR Green PCR Master Mix (Applied Biosystems) on a CFX96 real-time PCR detection system (BioRad). Reactions were performed in duplicate with the primer sequences listed in Table S6. RNA expression was normalized to that of the housekeeping gene *Gapdh*. FusionRed DNA copy number was normalized to that of genomic Gapdh.

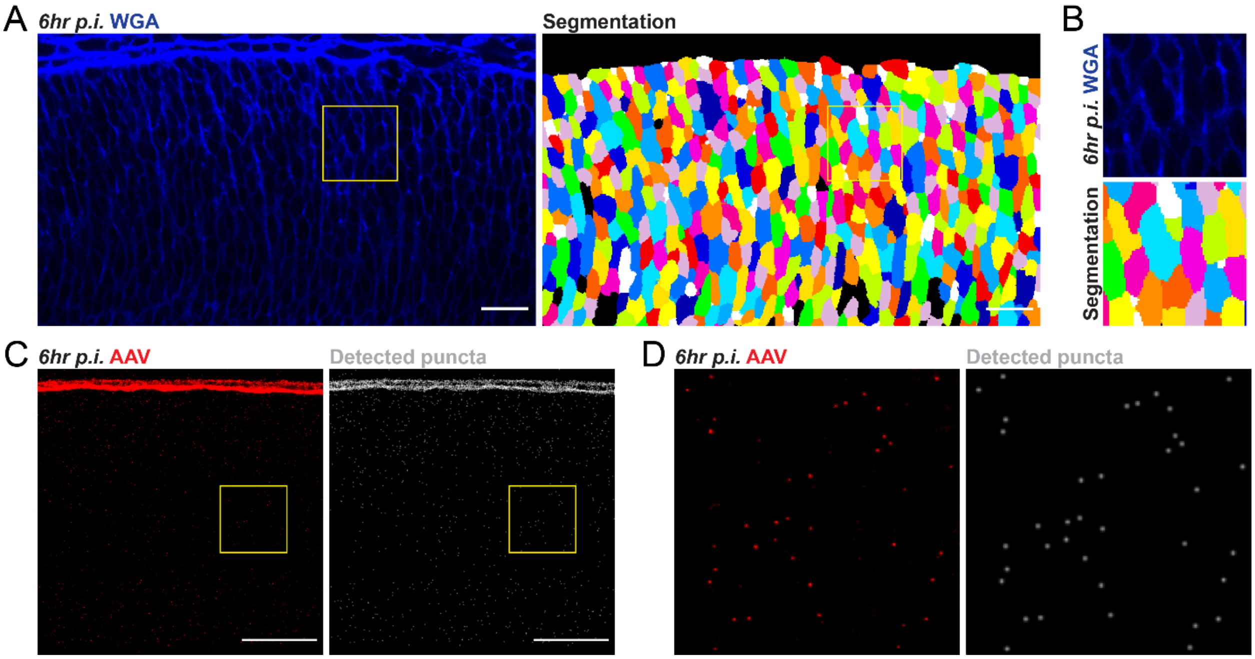

Figure S1. Automated cell segmentation and puncta detection.

(**A**, **B**) Low (A) and high (B) magnification images of cell membranes in the retina visualized with WGA and the corresponding output following automated cell segmentation.[2] Different colors indicate boundaries of adjoining cell bodies. Scale bars, 20 µm. (**C**, **D**) Low (C) and high (D) magnification images of fluorescent puncta at 6 hours after subretinal injection of ~2.5 x 10^8 vg of AAV8-CMV-GFP and the corresponding puncta following automated puncta detection.[3] Scale bars, 50 µm.

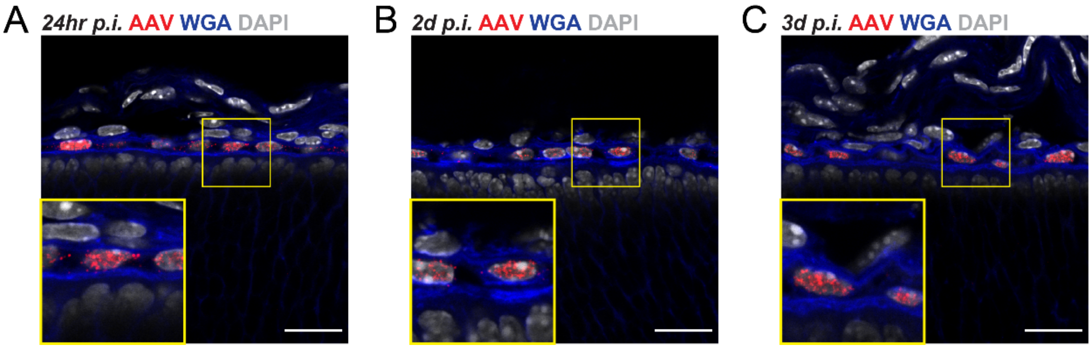

Figure S2. Nuclear localization of AAV genomes in the RPE.

(**A-C**) Low and high magnification images of AAV genomes in RPE nuclei at 24 hours (A), two days (B), and three days (C) after subretinal injection of ~2.5 x 10^8 vg of AAV8-CMV-GFP. Nuclei were labeled with DAPI. Scale bars, 20 µm.

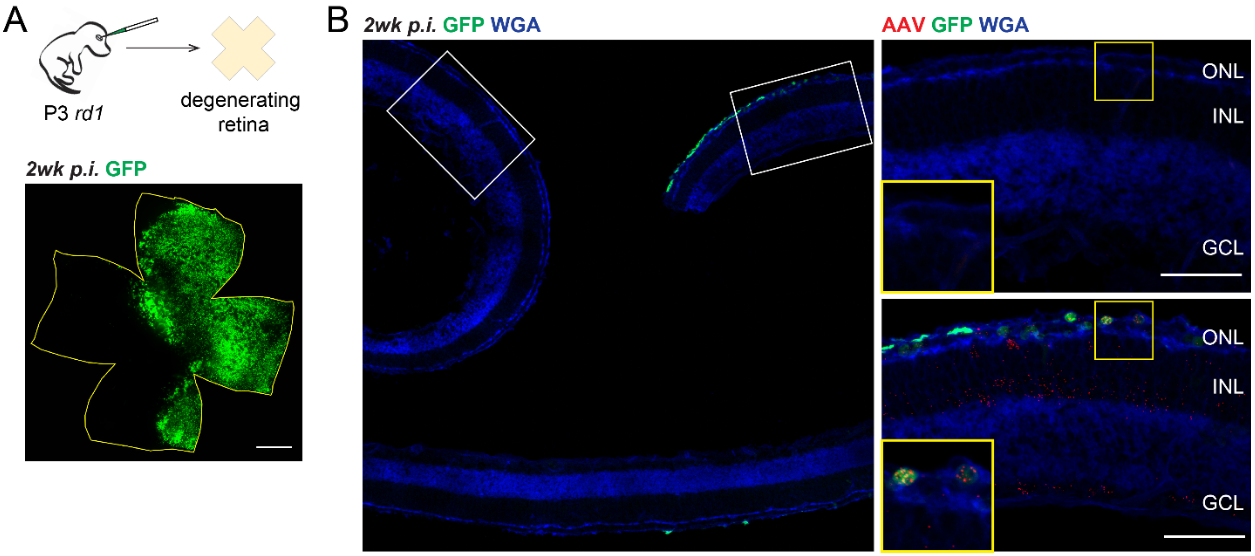

Figure S3. AAV genomes in the *rd1* disease model.

(**A**) Partial infection of a degenerating *rd1* retina after subretinal injection of ~2.5 x 10^8 vg of AAV8-CMV-GFP on postnatal day 3. Boundaries of the flat-mounted retina are outlined in yellow. Scale bar, 1 mm. (**B**) Low and high magnification images of AAV8-CMV-GFP genomes in GFP-negative (top) and GFP-positive (bottom) regions of the retina. Only a thin layer of photoreceptors remains in the ONL at this time point due to severe retinal degeneration. Scale bars, 50 µm. INL, inner nuclear layer; GCL, ganglion cell layer.

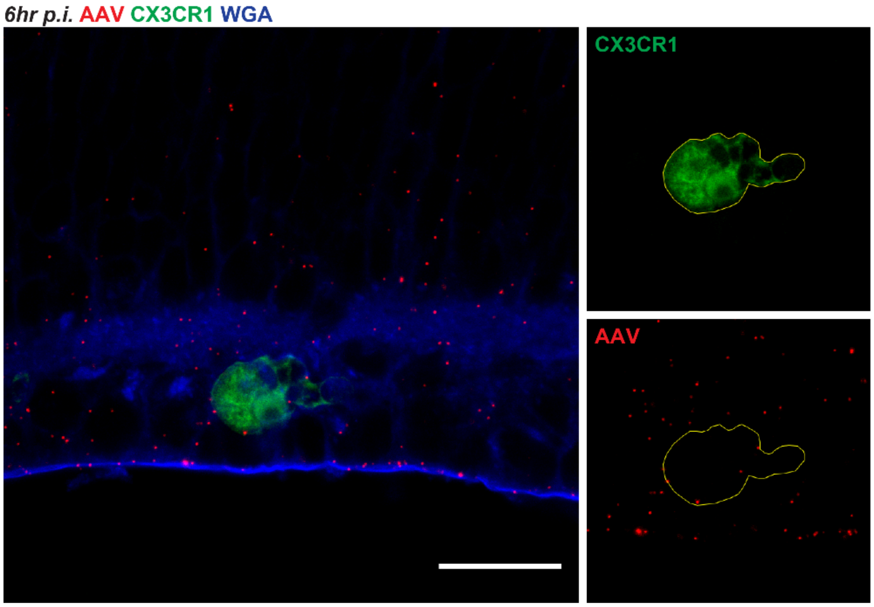

Figure S4. AAV genomes in retinal microglia at 6 hours post-injection.

Example of a CX3CR1-positive microglia with intracellular AAV genomes at 6 hours after subretinal injection of ~2.5 x 10^8 vg of AAV8-CMV-GFP. Cell boundaries of the microglia are outlined in yellow. Scale bar, 20 µm.

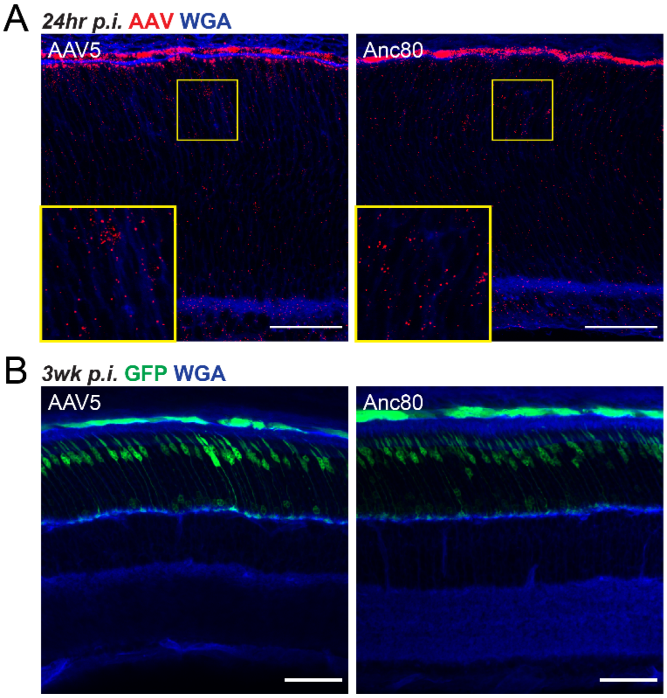

Figure S5. Distribution of AAV5-CMV-GFP and AAVAnc80-CMV-GFP in the retina.

(**A**) Low and high magnification images of retinas at 24 hours after subretinal injection of ~2.5 x 10^8 vg of AAV5-CMV-GFP or AAVAnc80-CMV-GFP. Scale bars, 50 µm. (**B**) Representative sections of the eye at 3 weeks after subretinal injection of ~2.5 x 10^8 vg of AAV5-CMV-GFP or AAVAnc80-CMV-GFP. Both vectors resulted in GFP expression in rods, cones, and the RPE. Scale bars, 50 µm.

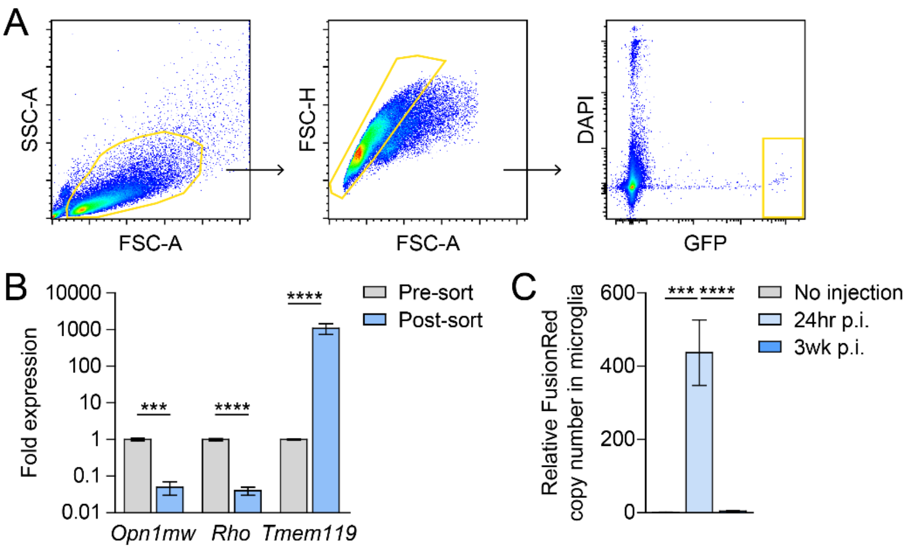

Figure S6. Detection of AAV genomes in retinal microglia by qPCR.

(**A**) Representative fluorescence-activated cell sorting (FACS) gating to isolate GFP-positive microglia from CX3CR1$^{GFP/+}$ retinas. (**B**) RNA expression of cone- (*Opn1mw*), rod- (*Rho*), and microglia- (*Tmem119*)[4] specific genes before and after sorting from three-week-old CX3CR1$^{GFP/+}$ retinas. Fold changes are relative to unsorted cells. (**C**) Copy number of FusionRed in sorted microglia at 24 hours or 3 weeks after subretinal injection of ~2.5 x 10^8 vg of AAV8-CMV-H2B-FusionRed. Values are relative to microglia from uninjected postnatal day 1 retinas. n = 3-4 animals per group for (B) and (C). Data shown are mean ± SEM. *** P<0.001, **** P<0.0001 by two-tailed Student's t-test.

Table S1. SABER-FISH probe sequences for AAV8-CMV-GFP.

ID	*Sequence*
CMVGFP_27_1	CAGAGAGGGAGTGGCCAACTCCATCACTAGGGGTTCTTTCATCATCAT
CMVGFP_27_2	AGTTAATGATTAACCCGCCATGCTACTTATCTACGTAGCCATTTCATCATCAT
CMVGFP_27_3	AAAATTAGTCAGCCATGAGCTTGGCCCATTGCATACTTTCATCATCAT
CMVGFP_27_4	TGTACATTTATATTGGCTCATGTCCAACATTACCGCCATGTTTTCATCATCAT
CMVGFP_27_5	ATTACGGGGTCATTAGTTCATAGCCCATATATGGAGTTCCGTTTCATCATCAT
CMVGFP_27_6	CGTTACATAACTTACGGTAAATGGCCCGCCTGGCTGTTTCATCATCAT
CMVGFP_27_7	CCCCGCCCATTGACGTCAATAATGACGTATGTTCCCTTTCATCATCAT
CMVGFP_27_8	GTGGAGTATTTACGGTAAACTGCCCACTTGGCAGTATTTCATCATCAT
CMVGFP_27_9	CCCCTATTGACGTCAATGACGGTAAATGGCCCGCCTTTTCATCATCAT
CMVGFP_27_10	GGCATTATGCCCAGTACATGACCTTATGGGACTTTCCTTTCATCATCAT
CMVGFP_27_11	TGGCAGTACATCTACGTATTAGTCATCGCTATTACCATGGTTTTCATCATCAT
CMVGFP_27_12	GATGCGGTTTTGGCAGTACATCAATGGGCGTGGATATTTCATCATCAT
CMVGFP_27_13	CGGTTTGACTCACGGGGATTTCCAAGTCTCCACCCCTTTCATCATCAT
CMVGFP_27_14	ATTGACGTCAATGGGAGTTTGTTTTGGCACCAAAATCATTTCATCATCAT
CMVGFP_27_15	ACGGGACTTTCCAAAATGTCGTAACAACTCCGCCCCTTTCATCATCAT
CMVGFP_27_16	CAAATGGGCGGTAGGCGTGTACGGTGGGAGGTCTATATTTTCATCATCAT
CMVGFP_27_17	AAGCAGAGCTCGTTTAGTGAACCGTCAGATCGCCTGTTTCATCATCAT
CMVGFP_27_18	CGATCCAGCCTCCCCTCGAAGCTGATCCTGAGAACTTTTCATCATCAT
CMVGFP_27_19	TCAGGGTGAGTCTATGGGACCCTTGATGTTTTCTTTCTTTCATCATCAT
CMVGFP_27_20	ATGGTTAAGTTCATGTCATAGGAAGGGGAGAAGTAACAGGGTTTCATCATCAT
CMVGFP_27_21	TCAGGGCAATAATGATACAATGTATCATGCCTCTTTGCACCTTTCATCATCAT
CMVGFP_27_22	TCATATTGCTAATAGCAGCTACAATCCAGCTACCATTCTGCTTTCATCATCAT
CMVGFP_27_23	TTTTATGGTTGGGATAAGGCTGGATTATTCTGAGTCCAAGCTTTCATCATCAT
CMVGFP_27_24	GGCCCTTTTGCTAATCATGTTCATACCTCTTATCTTCCTCCTTTCATCATCAT
CMVGFP_27_25	CTGGTCTGTGTGCTGGCCCATCACTTTGGCAAAGAATTTCATCATCAT
CMVGFP_27_26	GCTACCCCGACCACATGAAGCAGCACGACTTCTTCATTTCATCATCAT
CMVGFP_27_27	CGAAGGCTACGTCCAGGAGCGCACCATCTTCTTCAATTTCATCATCAT
CMVGFP_27_28	GTGAACCGCATCGAGCTGAAGGGCATCGACTTCAAGTTTCATCATCAT
CMVGFP_27_29	CGGCAACATCCTGGGGCACAAGCTGGAGTACAACTATTTCATCATCAT
CMVGFP_27_30	CAACAGCCACAACGTCTATATCATGGCCGACAAGCATTTCATCATCAT
CMVGFP_27_31	GAAGAACGGCATCAAGGTGAACTTCAAGATCCGCCATTTCATCATCAT
CMVGFP_27_32	CTGAGCAAAGACCCCAACGAGAAGCGCGATCACATGTTTCATCATCAT
CMVGFP_27_33	CCGGGATCACTCTCGGCATGGACGAGCTGTACAAGTATTTCATCATCAT
CMVGFP_27_34	AAGCGGCCGCTCTAGAGGATCCAAGCTTATCGATACTTTCATCATCAT
CMVGFP_27_35	CCAGATCTAATTCACCCCACCAGTGCAGGCTGCCTATTTCATCATCAT
CMVGFP_27_36	CTGGTGTGGCTAATGCCCTGGCCCACAAGTATCACTTTTCATCATCAT
CMVGFP_27_37	AAGCTCGCTTTCTTGCTGTCCAATTTCTATTAAAGGTTCCTTTTCATCATCAT
CMVGFP_27_38	GGGGATATTATGAAGGGCCTTGAGCATCTGGATTCTTTTCATCATCAT
CMVGFP_27_39	ATATCTTAAACTCCATGAAAGAAGGTGAGGCTGCAAACAGCTTTCATCATCAT
CMVGFP_27_40	TAATGCACATTGGCAACAGCCCCTGATGCCTATGCCTTTCATCATCAT
CMVGFP_27_41	TTCATCCCTCAGAAAAGGATTCAAGTAGAGGCTTGATTTGGTTTCATCATCAT
CMVGFP_27_42	TTTAGCTGTCCTCATGAATGTCTTTTCACTACCCATTTGCTTTTCATCATCAT
CMVGFP_27_43	TATCCTGCATCTCTCAGCCTTGACTCCACTCAGTTCTTTCATCATCAT
CMVGFP_27_44	TCTTGCTTAGAGATACCACCTTTCCCCTGAAGTGTTCCTTTCATCATCAT
CMVGFP_27_45	TTCCATGTTTTACGGCGAGATGGTTTCTCCTCGCCTTTTCATCATCAT
CMVGFP_27_46	GGCCACTCAGCCTTAGTTGTCTCTGTTGTCTTATAGAGGTTTCATCATCAT
CMVGFP_27_47	CCCCACTCACAGTGACCCGGAATCCCTCGACATCTATTTCATCATCAT

Table S2. SABER-FISH probe sequences for 5' and 3' halves of AAV8-CMV-GFP.

ID	*Sequence*
AAV-5'_28_1	CAGAGAGGGAGTGGCCAACTCCATCACTAGGGGTTCTTTCAACTTAAC
AAV-5'_28_2	AGTTAATGATTAACCCGCCATGCTACTTATCTACGTAGCCATTTCAACTTAAC
AAV-5'_28_3	AAAATTAGTCAGCCATGAGCTTGGCCCATTGCATACTTTCAACTTAAC
AAV-5'_28_4	TGTACATTTATATTGGCTCATGTCCAACATTACCGCCATGTTTTCAACTTAAC
AAV-5'_28_5	ATTACGGGGTCATTAGTTCATAGCCCATATATGGAGTTCCGTTTCAACTTAAC
AAV-5'_28_6	CGTTACATAACTTACGGTAAATGGCCCGCCTGGCTGTTTCAACTTAAC
AAV-5'_28_7	CCCCGCCCATTGACGTCAATAATGACGTATGTTCCCTTTCAACTTAAC
AAV-5'_28_8	GTGGAGTATTTACGGTAAACTGCCCACTTGGCAGTATTTCAACTTAAC
AAV-5'_28_9	CCCCTATTGACGTCAATGACGGTAAATGGCCCGCCTTTTCAACTTAAC
AAV-5'_28_10	GGCATTATGCCCAGTACATGACCTTATGGGACTTTCCTTTCAACTTAAC
AAV-5'_28_11	TGGCAGTACATCTACGTATTAGTCATCGCTATTACCATGGTTTTCAACTTAAC
AAV-5'_28_12	GATGCGGTTTTGGCAGTACATCAATGGGCGTGGATATTTCAACTTAAC
AAV-5'_28_13	CGGTTTGACTCACGGGGATTTCCAAGTCTCCACCCCTTTCAACTTAAC
AAV-5'_28_14	ATTGACGTCAATGGGAGTTTGTTTTGGCACCAAAATCATTTCAACTTAAC
AAV-5'_28_15	ACGGGACTTTCCAAAATGTCGTAACAACTCCGCCCCTTTCAACTTAAC
AAV-5'_28_16	CAAATGGGCGGTAGGCGTGTACGGTGGGAGGTCTATATTTTCAACTTAAC
AAV-5'_28_17	AAGCAGAGCTCGTTTAGTGAACCGTCAGATCGCCTGTTTCAACTTAAC
AAV-5'_28_18	CGATCCAGCCTCCCCTCGAAGCTGATCCTGAGAACTTTTCAACTTAAC
AAV-5'_28_19	TCAGGGTGAGTCTATGGGACCCTTGATGTTTTCTTTCTTTCAACTTAAC
AAV-5'_28_20	ATGGTTAAGTTCATGTCATAGGAAGGGGAGAAGTAACAGGGTTTCAACTTAAC
AAV-5'_28_21	TCAGGGCAATAATGATACAATGTATCATGCCTCTTTGCACCTTTCAACTTAAC
AAV-5'_28_22	TCATATTGCTAATAGCAGCTACAATCCAGCTACCATTCTGCTTTCAACTTAAC
AAV-5'_28_23	TTTTATGGTTGGGATAAGGCTGGATTATTCTGAGTCCAAGCTTTCAACTTAAC
AAV-5'_28_24	GGCCCTTTTGCTAATCATGTTCATACCTCTTATCTTCCTCCTTTCAACTTAAC
AAV-3'_27_25	CTGGTCTGTGTGCTGGCCCATCACTTTGGCAAAGAATTTCATCATCAT
AAV-3'_27_26	GCTACCCCGACCACATGAAGCAGCACGACTTCTTCATTTCATCATCAT
AAV-3'_27_27	CGAAGGCTACGTCCAGGAGCGCACCATCTTCTTCAATTTCATCATCAT
AAV-3'_27_28	GTGAACCGCATCGAGCTGAAGGGCATCGACTTCAAGTTTCATCATCAT
AAV-3'_27_29	CGGCAACATCCTGGGGCACAAGCTGGAGTACAACTATTTCATCATCAT
AAV-3'_27_30	CAACAGCCACAACGTCTATATCATGGCCGACAAGCATTTCATCATCAT
AAV-3'_27_31	GAAGAACGGCATCAAGGTGAACTTCAAGATCCGCCATTTCATCATCAT
AAV-3'_27_32	CTGAGCAAAGACCCCAACGAGAAGCGCGATCACATGTTTCATCATCAT
AAV-3'_27_33	CCGGGATCACTCTCGGCATGGACGAGCTGTACAAGTATTTCATCATCAT
AAV-3'_27_34	AAGCGGCCGCTCTAGAGGATCCAAGCTTATCGATACTTTCATCATCAT
AAV-3'_27_35	CCAGATCTAATTCACCCCACCAGTGCAGGCTGCCTATTTCATCATCAT
AAV-3'_27_36	CTGGTGTGGCTAATGCCCTGGCCCACAAGTATCACTTTTCATCATCAT
AAV-3'_27_37	AAGCTCGCTTTCTTGCTGTCCAATTTCTATTAAAGGTTCCTTTTCATCATCAT
AAV-3'_27_38	GGGGATATTATGAAGGGCCTTGAGCATCTGGATTCTTTTCATCATCAT
AAV-3'_27_39	ATATCTTAAACTCCATGAAAGAAGGTGAGGCTGCAAACAGCTTTCATCATCAT
AAV-3'_27_40	TAATGCACATTGGCAACAGCCCCTGATGCCTATGCCTTTCATCATCAT
AAV-3'_27_41	TTCATCCCTCAGAAAAGGATTCAAGTAGAGGCTTGATTTGGTTTCATCATCAT
AAV-3'_27_42	TTTAGCTGTCCTCATGAATGTCTTTTCACTACCCATTTGCTTTTCATCATCAT
AAV-3'_27_43	TATCCTGCATCTCTCAGCCTTGACTCCACTCAGTTCTTTCATCATCAT
AAV-3'_27_44	TCTTGCTTAGAGATACCACCTTTCCCCTGAAGTGTTCCTTTCATCATCAT
AAV-3'_27_45	TTCCATGTTTTACGGCGAGATGGTTTCTCCTCGCCTTTTCATCATCAT
AAV-3'_27_46	GGCCACTCAGCCTTAGTTGTCTCTGTTGTCTTATAGAGGTTTCATCATCAT
AAV-3'_27_47	CCCCACTCACAGTGACCCGGAATCCCTCGACATCTATTTCATCATCAT

Table S3. SABER-FISH probe sequences for AAV8-Best1-sCX3CL1.

ID	*Sequence*
Best1sCX3CL1_28_1	TCCTTTTCAGATAAGGGCACTGAGGCTGAGAGAGGATTTCAACTTAAC
Best1sCX3CL1_28_2	ACCAGAAACCAGGACTGTTGACTGCAGCCCGGTATTTTTCAACTTAAC
Best1sCX3CL1_28_3	CATTCTTTCCATAGCCCACAGGGCTGTCAAAGACCCTTTCAACTTAAC
Best1sCX3CL1_28_4	AGGGCCTAGTCAGAGGCTCCTCCTTCCTGGAGAGTTTTTCAACTTAAC
Best1sCX3CL1_28_5	AACTCTCTCTGCAAGGCCTCAGGGGTCAGAACACTGTTTCAACTTAAC
Best1sCX3CL1_28_6	GTGGAGCAGATCCTTTAGCCTCTGGATTTTAGGGCCTTTCAACTTAAC
Best1sCX3CL1_28_7	ATGGTAGAGGGGGTGTTGCCCTAAATTCCAGCCCTGTTTCAACTTAAC
Best1sCX3CL1_28_8	GTCTCAGCCCAACACCCTCCAAGAAGAAATTAGAGGTTTCAACTTAAC
Best1sCX3CL1_28_9	AGGCTGTGCTAGCCGTTGCTTCTGAGCAGATTACAATTTCAACTTAAC
Best1sCX3CL1_28_10	GAAGGGACTAAGACAAGGACTCCTTTGTGGAGGTCCTTTCAACTTAAC
Best1sCX3CL1_28_11	TAGTCGCCAGACCCAGATCCTGCAGAAGTTGGTCGTTTTCAACTTAAC
Best1sCX3CL1_28_12	GAGGCACTGGGCAGGTAAGTATCAAGGTTACAAGACTTTCAACTTAAC
Best1sCX3CL1_28_13	AGGTTTAAGGAGACCAATAGAAACTGGGCTTGTCGATTTCAACTTAAC
Best1sCX3CL1_28_14	GACAGAGAAGACTCTTGCGTTTCTGATAGGCACCTATTTCAACTTAAC
Best1sCX3CL1_28_15	TTGGTCTTACTGACATCCACTTTGCCTTTCTCTCCATTTCAACTTAAC
Best1sCX3CL1_28_16	TTCTTCCATTTGTGTACTCTGCTGCCGGGTCAGCACTTTCAACTTAAC
Best1sCX3CL1_28_17	CTCGGCATGACGAAATGCGAAATCATGTGCGACAAGTTTCAACTTAAC
Best1sCX3CL1_28_18	TCACGAATCCCAGTGGCTTTGCTCATCCGCTATCAGTTTCAACTTAAC
Best1sCX3CL1_28_19	ATTGTCCTGGAGACGACACAGCACAGACGCTTCTGTTTTCAACTTAAC
Best1sCX3CL1_28_20	AAGGAGAAATGGGTCCAAGACGCCATGAAGCATCTGTTTCAACTTAAC
Best1sCX3CL1_28_21	GATCACCAGGCTGCTGCCCTCACTAAAAATGGTGGCTTTCAACTTAAC
Best1sCX3CL1_28_22	AAGTTTGAGAAGCGGGTGGACAATGTGACACCTGGGTTTCAACTTAAC
Best1sCX3CL1_28_23	TGACAAAGCCTGAATCCGCCACATTGGAAGACCTTGTTTCAACTTAAC
Best1sCX3CL1_28_24	CTTTGGAACTGACTACTATTTCCCAGGAGGCCAGGGTTTCAACTTAAC
Best1sCX3CL1_28_25	AGCAGTGACCGGATCATCTCTCTCAACTTCCGAGGCTTTCAACTTAAC
Best1sCX3CL1_28_26	TTACGGCTAAGCCTCAGAGCATTGGAAGTTTTGAGGTTTCAACTTAAC
Best1sCX3CL1_28_27	CGTTTGGCCGAGTCCTGCTGTCTACCAATCTGGATCTTTCAACTTAAC
Best1sCX3CL1_28_28	TAGCTCCTGGGCTGAGGAAAAAGCTACTGAGTCCCCTTTCAACTTAAC
Best1sCX3CL1_28_29	CTCCACTACAGCCCCATCTCCTCAGGTGTCCACTACTTTCAACTTAAC
Best1sCX3CL1_28_30	TTCACCTTCAACCCCAGAGGAAAATGTTGGGTCCGATTTCAACTTAAC
Best1sCX3CL1_28_31	TCCAGGGACAGGACCTCAGTCCAGAGAAGTCTCTAGTTTCAACTTAAC
Best1sCX3CL1_28_32	ACCCAGTTCATACTGATAATTTCCAGGAGAGGGGGCTTTCAACTTAAC
Best1sCX3CL1_28_33	AGCCAGGCTCCTAAGATAGAGGAACCCATCCATGCCTTTCAACTTAAC
Best1sCX3CL1_28_34	ACTGCAGATCCCCAGAAACTGAGTGTGCTTATCACTTTTCAACTTAAC
Best1sCX3CL1_28_35	ACACCCAGGCAGCCACATGAAAGCTTGGATCCAATCTTTCAACTTAAC
Best1sCX3CL1_28_36	CGCTATGTGGATACGCTGCTTTAATGCCTTTGTATCATGCTTTCAACTTAAC
Best1sCX3CL1_28_37	AATCCTGGTTGCTGTCTCTTTATGAGGAGTTGTGGCTTTCAACTTAAC
Best1sCX3CL1_28_38	ACTGACAATTCCGTGGTGTTGTCGGGGAAATCATCGTTTCAACTTAAC
Best1sCX3CL1_28_39	CGCGTCTTCGAGATCTGCCTCGACTGTGCCTTCTAGTTTCAACTTAAC
Best1sCX3CL1_28_40	CCTGGAAGGTGCCACTCCCACTGTCCTTTCCTAATATTTCAACTTAAC
Best1sCX3CL1_28_41	TCGCATTGTCTGAGTAGGTGTCATTCTATTCTGGGGTTTCAACTTAAC
Best1sCX3CL1_28_42	AGGACAGCAAGGGGGAGGATTGGGAAGACAATAGCATTTCAACTTAAC
Best1sCX3CL1_28_43	ATGCTGGGGACTCGAGTTAAGGGCGAATTCCCGATATTTCAACTTAAC

Table S4. SABER-FISH probe sequences for AAV8-CMV-H2B-FusionRed.

ID	*Sequence*
CMVFusRed_28_1	CAGAGAGGGAGTGGCCAACTCCATCACTAGGGGTTCTTTCAACTTAAC
CMVFusRed_28_2	AGTTAATGATTAACCCGCCATGCTACTTATCTACGTAGCCATTTCAACTTAAC
CMVFusRed_28_3	AAAATTAGTCAGCCATGAGCTTGGCCCATTGCATACTTTCAACTTAAC
CMVFusRed_28_4	TGTACATTTATATTGGCTCATGTCCAACATTACCGCCATGTTTTCAACTTAAC
CMVFusRed_28_5	ATTACGGGGTCATTAGTTCATAGCCCATATATGGAGTTCCGTTTCAACTTAAC
CMVFusRed_28_6	CGTTACATAACTTACGGTAAATGGCCCGCCTGGCTGTTTCAACTTAAC
CMVFusRed_28_7	CCCCGCCCATTGACGTCAATAATGACGTATGTTCCCTTTCAACTTAAC
CMVFusRed_28_8	GTGGAGTATTTACGGTAAACTGCCCACTTGGCAGTATTTCAACTTAAC
CMVFusRed_28_9	CCCCTATTGACGTCAATGACGGTAAATGGCCCGCCTTTTCAACTTAAC
CMVFusRed_28_10	GGCATTATGCCCAGTACATGACCTTATGGGACTTTCCTTTCAACTTAAC
CMVFusRed_28_11	TGGCAGTACATCTACGTATTAGTCATCGCTATTACCATGGTTTTCAACTTAAC
CMVFusRed_28_12	GATGCGGTTTTGGCAGTACATCAATGGGCGTGGATATTTCAACTTAAC
CMVFusRed_28_13	CGGTTTGACTCACGGGGATTTCCAAGTCTCCACCCCTTTCAACTTAAC
CMVFusRed_28_14	ATTGACGTCAATGGGAGTTTGTTTTGGCACCAAAATCATTTCAACTTAAC
CMVFusRed_28_15	ACGGGACTTTCCAAAATGTCGTAACAACTCCGCCCCTTTCAACTTAAC
CMVFusRed_28_16	CAAATGGGCGGTAGGCGTGTACGGTGGGAGGTCTATATTTCAACTTAAC
CMVFusRed_28_17	AAGCAGAGCTCGTTTAGTGAACCGTCAGATCGCCTGTTTCAACTTAAC
CMVFusRed_28_18	CGATCCAGCCTCCCCTCGAAGCTGATCCTGAGAACTTTTCAACTTAAC
CMVFusRed_28_19	TCAGGGTGAGTCTATGGGACCCTTGATGTTTTCTTTCTTTCAACTTAAC
CMVFusRed_28_20	ATGGTTAAGTTCATGTCATAGGAAGGGGAGAAGTAACAGGGTTTCAACTTAAC
CMVFusRed_28_21	TCAGGGCAATAATGATACAATGTATCATGCCTCTTTGCACCTTTCAACTTAAC
CMVFusRed_28_22	TCATATTGCTAATAGCAGCTACAATCCAGCTACCATTCTGCTTTCAACTTAAC
CMVFusRed_28_23	TTTTATGGTTGGGATAAGGCTGGATTATTCTGAGTCCAAGCTTTCAACTTAAC
CMVFusRed_28_24	GGCCCTTTTGCTAATCATGTTCATACCTCTTATCTTCCTCCTTTCAACTTAAC
CMVFusRed_28_25	GAGCTCAAGCTTCGAATTCTGCAGTCGACGGTACCGTTTCAACTTAAC
CMVFusRed_28_26	CAAGCGCAGCCGCAAGGAGAGCTATTCCATCTATGTTTCAACTTAAC
CMVFusRed_28_27	GTACAAGGTTCTGAAGCAGGTCCACCCTGACACCGGTTTCAACTTAAC
CMVFusRed_28_28	CATTTCGTCCAAGGCCATGGGCATCATGAATTCGTTTTTCAACTTAAC
CMVFusRed_28_29	ATTACAACAAGCGCTCGACCATCACCTCCAGGGAGATTTCAACTTAAC
CMVFusRed_28_30	ATCACCAAGTACACCAGCGCTAAGGATCCACCGGTCTTTCAACTTAAC
CMVFusRed_28_31	GCCACCATGGTGAGCGAGCTGATTAAGGAGAACATGTTTCAACTTAAC
CMVFusRed_28_32	CCCATGAAGCTGTACATGGAGGGCACCGTGAACAACTTTCAACTTAAC
CMVFusRed_28_33	ACATCCTGGCTACCAGCTTCATGTACGGCAGCAGAATTTCAACTTAAC
CMVFusRed_28_34	ACTTCTTTAAGCAGTCCTTCCCTGAGGGCTTCACATTTTCAACTTAAC
CMVFusRed_28_35	TCATCTACAACGTCAAGGTTAGAGGGGTGAACTTCCTTTCAACTTAAC
CMVFusRed_28_36	CAGCCAACGGCCCTGTGATGCAGAAGAAAACACTCGTTTCAACTTAAC
CMVFusRed_28_37	GCCACCTGATCTGCAACCTTGAGACCACATACAGATTTCAACTTAAC
CMVFusRed_28_38	GGCGTCTACAACGTGGACCACAGACTGGAAAGAATCTTTCAACTTAAC
CMVFusRed_28_39	CCAGATCTAATTCACCCCACCAGTGCAGGCTGCCTATTTCAACTTAAC
CMVFusRed_28_40	CTGGTGTGGCTAATGCCCTGGCCCACAAGTATCACTTTTCAACTTAAC
CMVFusRed_28_41	AAGCTCGCTTTCTTGCTGTCCAATTTCTATTAAAGGTTCCTTTTCAACTTAAC
CMVFusRed_28_42	GGGGATATTATGAAGGGCCTTGAGCATCTGGATTCTTTTCAACTTAAC
CMVFusRed_28_43	ATATCTTAAACTCCATGAAAGAAGGTGAGGCTGCAAACAGCTTTCAACTTAAC
CMVFusRed_28_44	TAATGCACATTGGCAACAGCCCCTGATGCCTATGCCTTTCAACTTAAC
CMVFusRed_28_45	TTCATCCCTCAGAAAAGGATTCAAGTAGAGGCTTGATTTGGTTTCAACTTAAC
CMVFusRed_28_46	TTTAGCTGTCCTCATGAATGTCTTTTCACTACCCATTTGCTTTTCAACTTAAC
CMVFusRed_28_47	TATCCTGCATCTCTCAGCCTTGACTCCACTCAGTTCTTTCAACTTAAC
CMVFusRed_28_48	TCTTGCTTAGAGATACCACCTTTCCCCTGAAGTGTTCCTTTCAACTTAAC
CMVFusRed_28_49	TTCCATGTTTTACGGCGAGATGGTTTCTCCTCGCCTTTTCAACTTAAC
CMVFusRed_28_50	GGCCACTCAGCCTTAGTTGTCTCTGTTGTCTTATAGAGGTTTCAACTTAAC
CMVFusRed_28_51	CCCCACTCACAGTGACCCGGAATCCCTCGACATCTATTTCAACTTAAC

Table S5. Catalytic hairpin and fluorescent oligonucleotide sequences.

ID	*Sequence*
Hairpin_27	ACATCATCATGGGCCTTTTGGCCCATGATGATGTATGATGATGTTTTTTT
Hairpin_28	ACAACTTAACGGGCCTTTTGGCCCGTTAAGTTGTGTTAAGTTGTTTTTTT
Fluor_27	/5ATTO550N/TTATGATGATGTATGATGATGT
Fluor_28	/5ATTO633N/TTGTTAAGTTGTGTTAAGTTGT

Table S6. qPCR Primers.

Target	*5' Sequence*	*3' Sequence*
FusionRed (DNA)	CTCCACCGAGACGATGTACC	TGAGGTTCGTAGCGGGTTTC
Gapdh (DNA)	GGTTGTCTCCTGCGACTTCA	CAGGTTTCCCATCCCCACAT
Gapdh (RNA)	AGGTCGGTGTGAACGGATTTG	TGTAGACCATGTAGTTGAGGTCA
Opn1mw (RNA)	ATGGCCCAAAGGCTTACAGG	CCACAAGAATCATCCAGGTGC
Rho (RNA)	CCCTTCTCCAACGTCACAGG	TGAGGAAGTTGATGGGGAAGC
Tmem119 (RNA)	CCTACTCTGTGTCACTCCCG	CACGTACTGCCGGAAGAAATC

SUPPLEMENTAL REFERENCES

1. Wang, S.K., Xue, Y., Rana, P., Hong, C.M. and Cepko, C.L. (2019). Soluble CX3CL1 gene therapy improves cone survival and function in mouse models of retinitis pigmentosa. *Proc. Natl. Acad. Sci. U. S. A.* 116: 10140–10149.

2. Mosaliganti, K.R., Noche, R.R., Xiong, F., Swinburne, I.A. and Megason, S.G. (2012). ACME: Automated Cell Morphology Extractor for Comprehensive Reconstruction of Cell Membranes. *PLoS Comput. Biol.* 8.

3. Kishi, J.Y., Lapan, S.W., Beliveau, B.J., West, E.R., Zhu, A., Sasaki, H.M., Saka, S.K., Wang, Y., Cepko, C.L. and Yin, P. (2019). SABER amplifies FISH: enhanced multiplexed imaging of RNA and DNA in cells and tissues. *Nat. Methods* 16: 533–544.

4. Bennett, M.L., Bennett, F.C., Liddelow, S.A., Ajami, B., Zamanian, J.L., Fernhoff, N.B., Mulinyawe, S.B., Bohlen, C.J., Adil, A., Tucker, A., *et al.* (2016). New tools for studying microglia in the mouse and human CNS. *Proc. Natl. Acad. Sci. U. S. A.* 113: E1738–E1746.
