# Supplementary material for: In Situ Detection of Adeno-associated Viral Vector Genomes with SABER-FISH
Source: Mol Ther Methods Clin Dev. 2020 Oct 10;19:376–86. doi: 10.1016/j.omtm.2020.10.003 (PMC7658570; doi:10.1016/j.omtm.2020.10.003)
Supplement: Document S2. Article plus Supplemental Information [file mmc2.pdf]

Molecular Therapy
Methods & Clinical Development
Original Article

In Situ Detection of Adeno-associated Viral Vector Genomes with SABER-FISH

Sean K. Wang,[1] Sylvain W. Lapan,[1] Christin M. Hong,[1] Tyler B. Krause,[1] and Constance L. Cepko[1,2]

[1]Departments of Genetics and Ophthalmology, Blavatnik Institute, Harvard Medical School, Boston, MA 02115, USA; [2]Howard Hughes Medical Institute, Chevy Chase, MD 20815, USA

Gene therapy with recombinant adeno-associated viral (AAV) vectors is a promising modality for the treatment of a variety of human diseases. Nonetheless, there remain significant gaps in our understanding of AAV vector biology, due in part to the lack of robust methods to track AAV capsids and genomes. In this study, we describe a novel application of signal amplification by exchange reaction fluorescence *in situ* hybridization (SABER-FISH) that enabled the visualization and quantification of individual AAV genomes after vector administration in mice. These genomes could be seen in retinal cells within 3 h of subretinal AAV delivery, were roughly full length, and correlated with vector expression in both photoreceptors and the retinal pigment epithelium. SABER-FISH readily detected AAV genomes in the liver and muscle following retro-orbital and intramuscular AAV injections, respectively, demonstrating its utility in different tissues. Using SABER-FISH, we also found that retinal microglia, a cell type deemed refractory to AAV transduction, are in fact efficiently infected by multiple AAV serotypes, but appear to degrade AAV genomes prior to nuclear localization. Our findings show that SABER-FISH can be used to visualize AAV genomes *in situ*, allowing for studies of AAV vector biology and the tracking of transduced cells following vector administration.

INTRODUCTION

Recombinant adeno-associated viral (AAV) vectors derived from adeno-associated viruses have become the vector of choice for gene therapy due to their long-term expression of transgenes and relative lack of pathogenicity.[1–4] Each AAV virion consists of a protein capsid surrounding a single-stranded DNA genome, which can be up to 4.7 kb.[3,5] Although capable of integration into host genomes,[6] AAV vectors in infected cells are thought to predominantly exist as extrachromosomal episomes in the nucleus.[7] These episomes are diluted in actively dividing cells, but they can be effectively maintained in post-mitotic cell types, such as those of the brain, retina, muscle, and heart.

In recent years, use of AAV vectors in patients has grown tremendously, highlighted by the advent of gene therapy treatments for Leber's congenital amaurosis and spinal muscular atrophy.[8,9] The translational potential of AAV vectors has now led to more than 200 phase I, II, and III clinical trials (https://clinicaltrials.gov). Despite these advances, there remain significant gaps in our understanding of AAV vectors, due in part to the lack of readily deployed methods to track vector capsids and genomes. For instance, it is still unknown in most cell types whether one or multiple transduction events are required for AAV transgenes to be expressed. Likewise, for vectors without a reporter gene, such as those administered in patients, it is often challenging to determine the identities of infected cells and distinguish them from uninfected cells in the same region. One strategy for tracking AAV vectors in animal models has been to tag capsid proteins with radioactive or fluorescent labels.[10–12] While this approach allows for the detection of vectors shortly after delivery, it can only provide information prior to the loss of viral capsid proteins and be applied to tissue samples that receive the labeled capsids. To address these concerns, an alternative is to visualize AAV vectors by targeting the vector genome with fluorescence *in situ* hybridization (FISH). However, the sensitivity of conventional FISH for DNA sequences below 5–10 kb is limited, a drawback that has precluded broader use of this technique in AAV and gene therapy research.

Signal amplification by exchange reaction (SABER) is a method that uses a strand-displacing polymerase and catalytic DNA hairpin to generate FISH probes containing long arrays of binding sites for fluorescent oligonucleotides.[13] Compared to probes used in conventional FISH, SABER-FISH probes result in up to several hundred-fold amplification of DNA and RNA signals, allowing for highly sensitive detection of nucleic acids within cells.[13] Herein, we describe a novel application of SABER-FISH that enables the visualization and quantification of individual AAV genomes *in situ*. These genomes can be detected in at least three different tissues that are currently targets of gene therapy, i.e., retina, liver, and muscle. In the eye, AAV expression was tracked and found to correlate with AAV genome counts. Furthermore, using SABER-FISH, we show that retinal microglia, a cell type thought to be resistant to infection with AAV vectors, are in fact efficiently transduced but appear to degrade AAV genomes prior to their translocation into the nucleus. Our findings illustrate the versatility of SABER-FISH as a tool to study AAV vectors with

Received 22 May 2020; accepted 7 October 2020;
https://doi.org/10.1016/j.omtm.2020.10.003.

Correspondence: Constance L. Cepko, Departments of Genetics and Ophthalmology, Blavatnik Institute, Harvard Medical School, 77 Avenue Louis Pasteur, NRB Room 360, Boston, MA 02115, USA.
E-mail: cepko@genetics.med.harvard.edu

 © 2020 The Author(s).
This is an open access article under the CC BY license (http://creativecommons.org/licenses/by/4.0/).

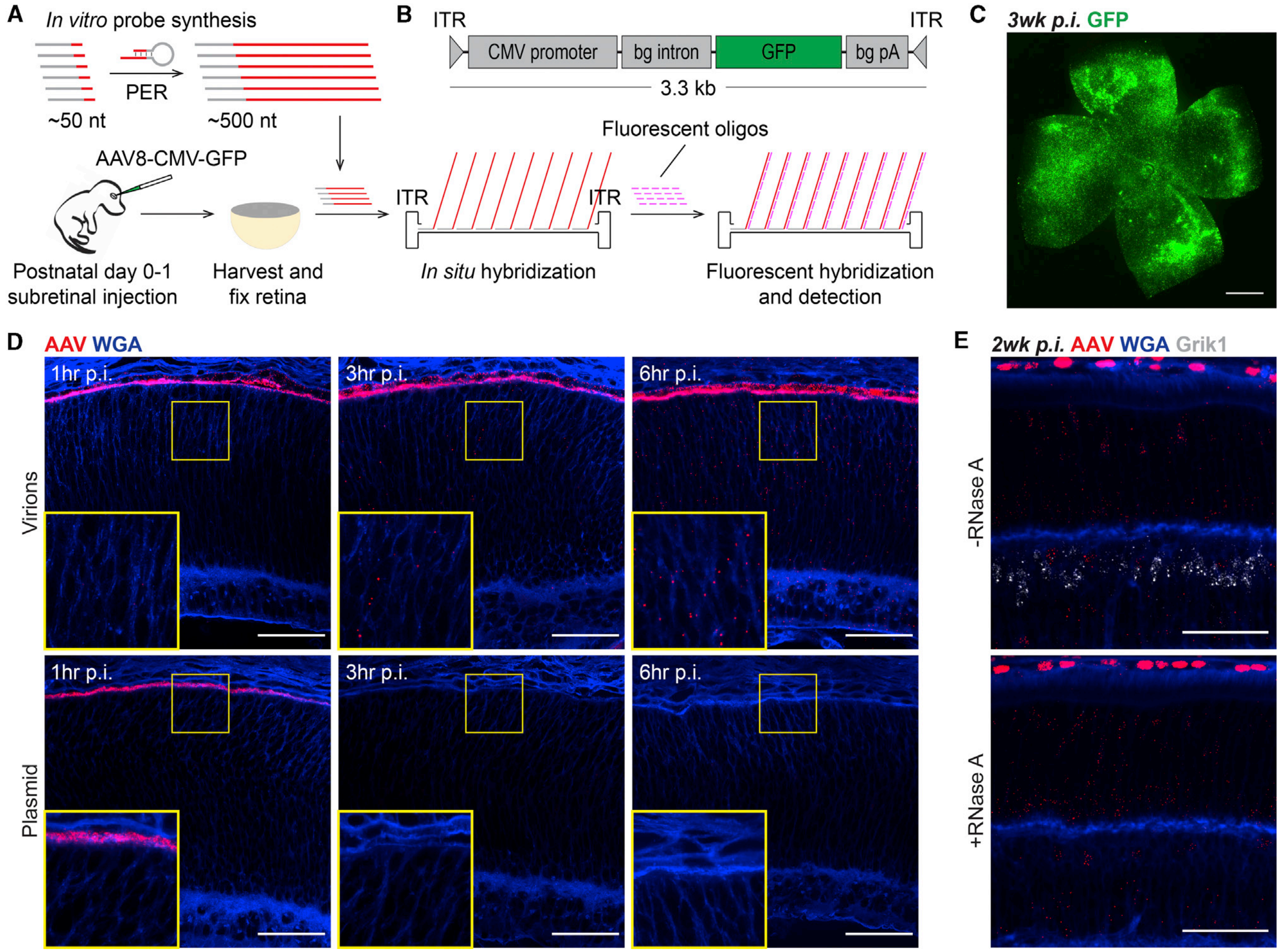

Figure 1. SABER-FISH Detects Fluorescent DNA Puncta after Subretinal Delivery of AAV Vectors

(A) Schematic of SABER-FISH protocol for detection of AAV genomes. SABER-FISH probes complementary to the negative-sense strand of the AAV8-CMV-GFP genome were synthesized *in vitro* and hybridized to fixed retinal tissue from mice that had been subretinally injected with the vector. Short fluorescent oligonucleotides were then hybridized to the primary SABER-FISH probes, allowing for signal amplification and fluorescent detection. (B) Schematic of AAV8-CMV-GFP vector design. (C) Representative image of a flat-mounted retina demonstrating pan-retinal GFP expression by 3 weeks after postnatal day 0–1 subretinal injection of ~0.25 μL of 1 × 10^{12} vector genomes (vg)/mL (~2.5 × 10^8 vg) of AAV8-CMV-GFP. Scale bar, 1 mm. (D) Low and high magnification images of retinas at 1, 3, or 6 h after subretinal injection of ~2.5 × 10^8 vg of AAV8-CMV-GFP or ~0.25 μL of 1 mg/mL of plasmid encoding the AAV8-CMV-GFP sequence. Cell membranes were labeled with WGA. Scale bars, 50 μm. (E) Simultaneous detection of AAV8-CMV-GFP puncta and *Grik1* mRNA in sections from the same retina with or without RNase A treatment. Scale bars, 50 μm. nt, nucleotide; PER, primer exchange reaction; ITR, inverted terminal repeat; bg, β-globin; pA, polyadenylation sequence.

the potential to expedite both preclinical and clinical efforts to develop AAV gene therapy.

RESULTS

SABER-FISH Detects Fluorescent DNA Puncta after Subretinal Delivery of AAV Vectors

SABER-FISH probes were first synthesized to target the genome of serotype 8 AAV (AAV8)-cytomegalovirus (CMV)-GFP, an AAV8 vector using the broadly active CMV promoter to drive expression of GFP (Figures 1A and 1B). AAV vectors possess a linear single-stranded DNA genome that converts to double-stranded DNA before generating mRNA and protein.[3] Probes were thus designed to recognize only the negative-sense strand of the AAV8-CMV-GFP genome to avoid binding of RNA transcribed from the vector. A total of 47 probes (Table S1) were made *in vitro* as previously described and hybridized to tissues infected with AAV8-CMV-GFP.[13] To model *in vivo* gene therapy, subretinal injections were performed. Delivery of AAV8-CMV-GFP by this route in mice resulted in a strong pan-retinal GFP signal by 3 weeks post-injection (p.i.) (Figure 1C), consistent with prior reports on the kinetics of AAV-mediated transgene expression.[14,15]

Probes were initially tested after subretinal injection of either AAV8-CMV-GFP virions, which were predicted to infect and enter retinal

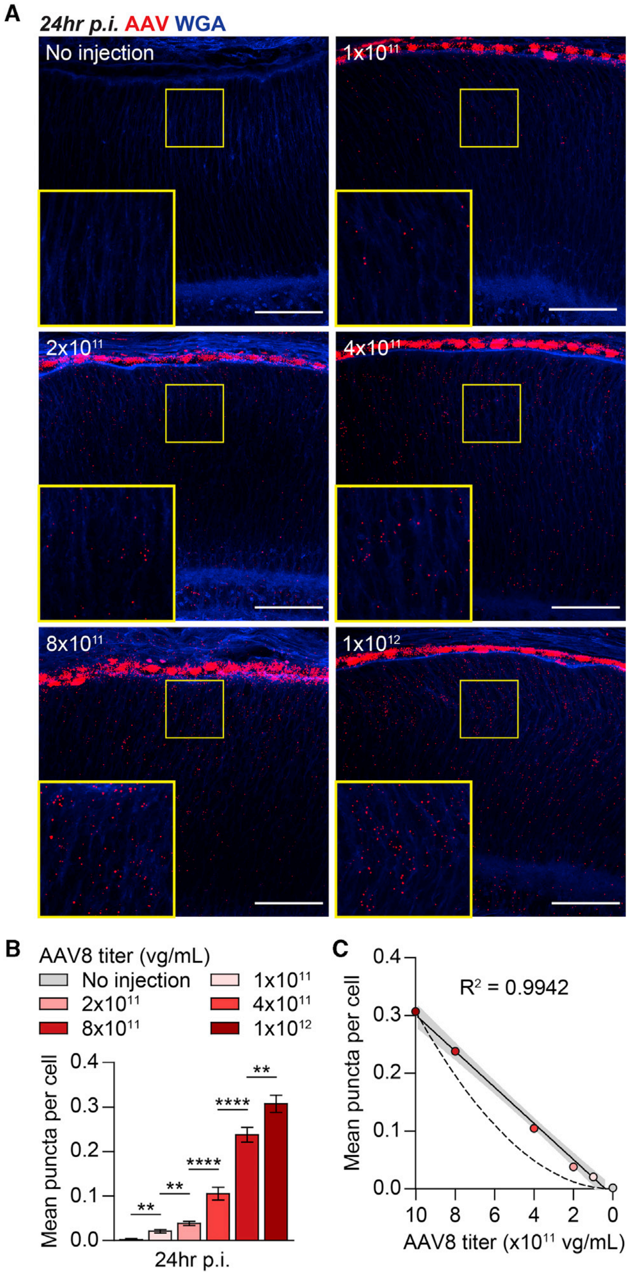

Figure 2. Quantification and Limiting Dilution Analysis of Fluorescent Puncta after Subretinal Delivery of AAV Vectors

(A) Low and high magnification images of retinas at 24 h after subretinal injection of ~0.25 μL of the indicated titers of AAV8-CMV-GFP. Scale bars, 50 μm. (B) Quantification of fluorescent puncta in cells of the retina with the conditions tested in (A). n = 477–1,733 cells from two to four animals per group. (C) Limiting dilution analysis of fluorescent puncta in retinas at 24 h p.i. based on the data shown in (B). Solid line depicts linear regression for the plotted data with 95% confidence interval shown in gray and goodness-of-fit measured by R^2. Dotted line depicts expected relationship if puncta count were to decrease by the square of the vector titer, as would occur if two independent AAV genomes in the same location were required to detect each punctum. Data shown are mean ± SEM. **p < 0.01, ****p < 0.0001 by two-tailed Student's t test with Bonferroni correction.

cells, or AAV8-CMV-GFP plasmid, which was predicted to remain in the subretinal space. Wheat germ agglutinin (WGA) was used to label cell membranes to enable the tracing of cellular boundaries. One hour after virion and plasmid injections, SABER-FISH detected many fluorescent puncta filling the subretinal space (Figure 1D, left panels). However, at 3 and 6 h p.i., puncta could be detected inside retinal cells only in the eyes that received virions (Figure 1D, center and right panels). To verify that these puncta corresponded to AAV genomes and not RNA transcribed from the vector or preexisting within the vector stock, tissues were treated with RNase A, which degrades RNA without affecting DNA. As a control, SABER-FISH was simultaneously used to detect *Grik1* mRNA found in bipolar interneurons. While treatment with RNase A eliminated the signal from *Grik1* mRNA, it had no effect on fluorescent puncta from AAV8-CMV-GFP (Figure 1E), consistent with the latter revealing viral DNA. SABER-FISH thus allows for the detection of viral DNA in eyes after subretinal injection of AAV vectors, with puncta visible inside retinal cells as early as 3 h p.i.

SABER-FISH Enables Visualization of Individual AAV Genomes in the Retina

Does each DNA punctum detected by SABER-FISH represent a single AAV genome or multiple genomes in close proximity? To distinguish between these possibilities, a series of five dilutions were made from the AAV8-CMV-GFP vector stock, which were then subretinally injected into mice. If each AAV genome generated a single fluorescent punctum, we reasoned that an x-fold decrease in AAV vector titer would likewise result in an x-fold decrease in the number of puncta; i.e., there would be a linear relationship between vector titer and puncta count. Conversely, if a group of y genomes in close proximity were needed to detect each punctum, the same x-fold drop in vector titer should produce an x^y-fold drop in puncta, assuming that the location of each genome was independently determined. Using methods previously described,[13,16] cell boundaries in the neural retina could be delineated in three dimensions based on their labeling with WGA (Figures S1A and S1B). Puncta in these images could then be computationally assigned to cells, facilitating automated quantification of puncta in the retina (Figures S1C and S1D). At 24 h p.i., puncta in retinal cells were significantly increased in eyes receiving higher titers of AAV8-CMV-GFP and were absent from uninjected controls (Figures 2A and 2B). The relationship between AAV vector titer and puncta count was strongly linear (Figure 2C, $R^2 = 0.9942$), consistent with the model of each DNA punctum visualized by SABER-FISH corresponding to a single AAV genome.

To assess the gross integrity of individual AAV genomes after subretinal delivery, 24 of the 47 probes for AAV8-CMV-GFP were modified to enable detection of the 5′ and 3′ halves of the vector genome in separate fluorescent channels (Table S2). Simultaneous detection of these partial genomes in the retina demonstrated colocalization in ~90% of cases at both 24 h and 2 weeks p.i. (Figures 3A and 3C), suggesting that most AAV genomes are roughly full length. As a comparison, another set of probes (Table S3) were synthesized to target a different vector, AAV8-Best1-sCX3CL1,[17] which was co-injected

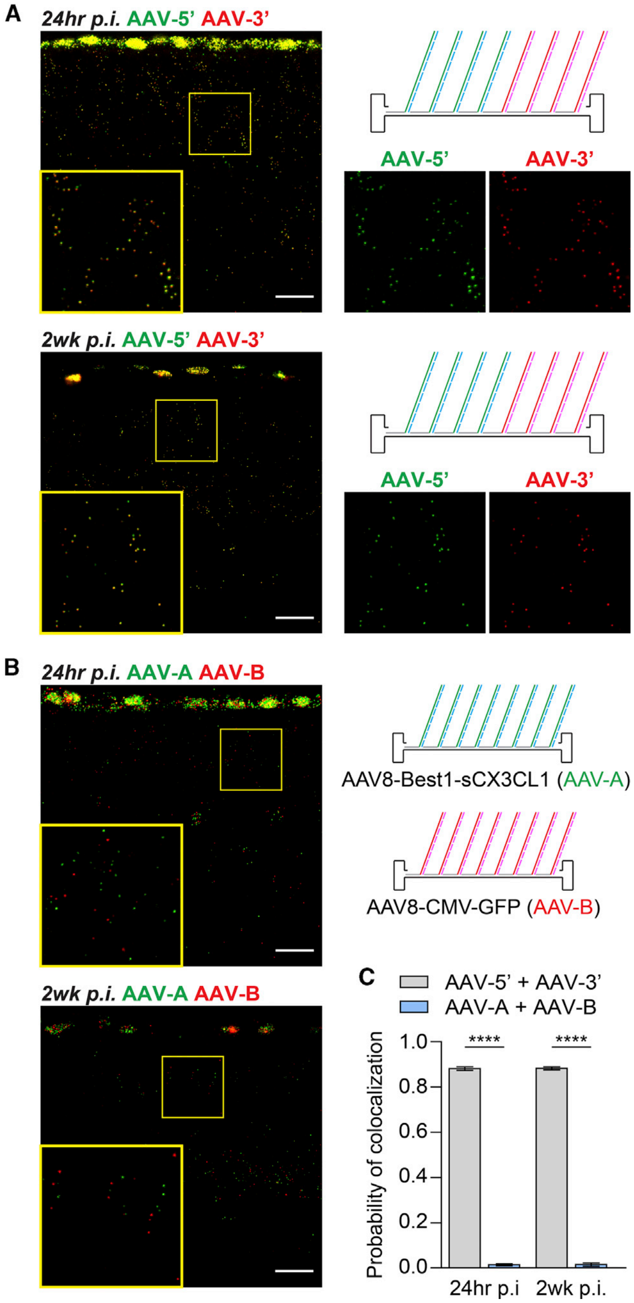

Figure 3. Detection of Partial AAV Genomes and Genomes from Different Vectors

(A) Low and high magnification images of the 5′ (AAV-5′, green) and 3′ (AAV-3′, red) halves of the AAV8-CMV-GFP genome in retinas at 24 h or 2 weeks after subretinal injection of ~2.5×10^8 vg of AAV8-CMV-GFP. Colocalized green and red puncta appear yellow. Scale bars, 20 μm. (B) Low and high magnification images of the AAV8-Best1-sCX3CL1 (AAV-A, green) and AAV8-CMV-GFP (AAV-B, red) genomes in retinas at 24 h or 2 weeks after subretinal injection of ~2.5×10^8 vg of each vector. Scale bars, 20 μm. (C) Probability of green and red colocalization for the combinations tested in (A) and (B), defined as the fraction of puncta in the retina that colocalized with puncta of the other color. n = 3–5 animals per group. Data shown are mean ± SEM. ****$p < 0.0001$ by two-tailed Student's t test.

with AAV8-CMV-GFP and analyzed at the same time points. Genomes from AAV8-Best1-sCX3CL1 and AAV8-CMV-GFP appeared randomly distributed in infected cells, with their corresponding puncta colocalizing in only ~1% of cases (Figures 3B and 3C). Taken together, these data support the ability of SABER-FISH to track individual AAV genomes from one or multiple vectors in a quantifiable manner.

AAV8-CMV-GFP Genome Number Correlates with GFP Expression

Following subretinal injection of AAV8-CMV-GFP in neonatal mice, GFP signal could be seen in the three cell types that line the injection site, including rod photoreceptors, cone photoreceptors, and the retinal pigment epithelium (RPE) (Figure 4A). For each of these cell types, we used SABER-FISH to ask how the number of AAV8-CMV-GFP genomes related to GFP expression. The outer nuclear layer (ONL) of the retina consists of the cell bodies of rods and cones, which can be identified by negative and positive immunostaining for cone arrestin, respectively. SABER-FISH in these cell bodies was therefore combined with cone arrestin immunostaining and detection of GFP to determine the distribution of AAV8-CMV-GFP genomes in GFP-negative and GFP-positive photoreceptors. For rods, 86.0% of GFP-negative cell bodies were devoid of AAV genomes, while 85.3% of GFP-positive cell bodies contained at least one genome, with a mean of 2.1 per cell (Figures 4B, 4D, and 4G). AAV8-CMV-GFP genomes in rods were thus highly predictive of GFP expression. For cones, AAV genomes were present in 91.2% of GFP-positive cell bodies, with a mean of 2.5 per cell (Figures 4B, 4E, and 4G). Alternatively, AAV genomes were also found in nearly half (48.0%) of the GFP-negative cone cell bodies, demonstrating that some cones do not express AAV8-CMV-GFP, at least to our level of detection.

To analyze RPE cells, AAV8-CMV-GFP genomes were quantified in RPE nuclei labeled with 4′,6-diamidino-2-phenylindole (DAPI), since the vast majority of genomes in the RPE localized to nuclei by 3 days p.i. (Figure S2). At 3 weeks p.i., GFP-negative RPE cells were relatively uncommon, although occasionally they could be seen among GFP-positive cells (Figure 4F). While all GFP-negative RPE nuclei examined had four or fewer copies of AAV8-CMV-GFP, most GFP-positive RPE nuclei had more than 20 genomes, with a mean of 29.9 per nucleus (Figures 4C and 4G). As in rods, AAV8-CMV-GFP genomes in RPE nuclei were therefore strongly associated with GFP expression. However, compared to the number in photoreceptors, these genomes were far more abundant in the RPE.

Among the rods, cones, and RPE cells that were GFP-positive, we asked whether those with more copies of AAV8-CMV-GFP exhibited higher levels of GFP fluorescence. For all three cell types, this was indeed the case (Figures 4H and 4I), supporting the notion that transduction with larger numbers of AAV genomes results in greater transgene expression. Finally, to test SABER-FISH in a model of human disease, subretinal injections of AAV8-CMV-GFP were performed in *rd1* mice, which undergo severe retinal degeneration within weeks of birth.[18] In regions of the retina with surviving GFP-positive

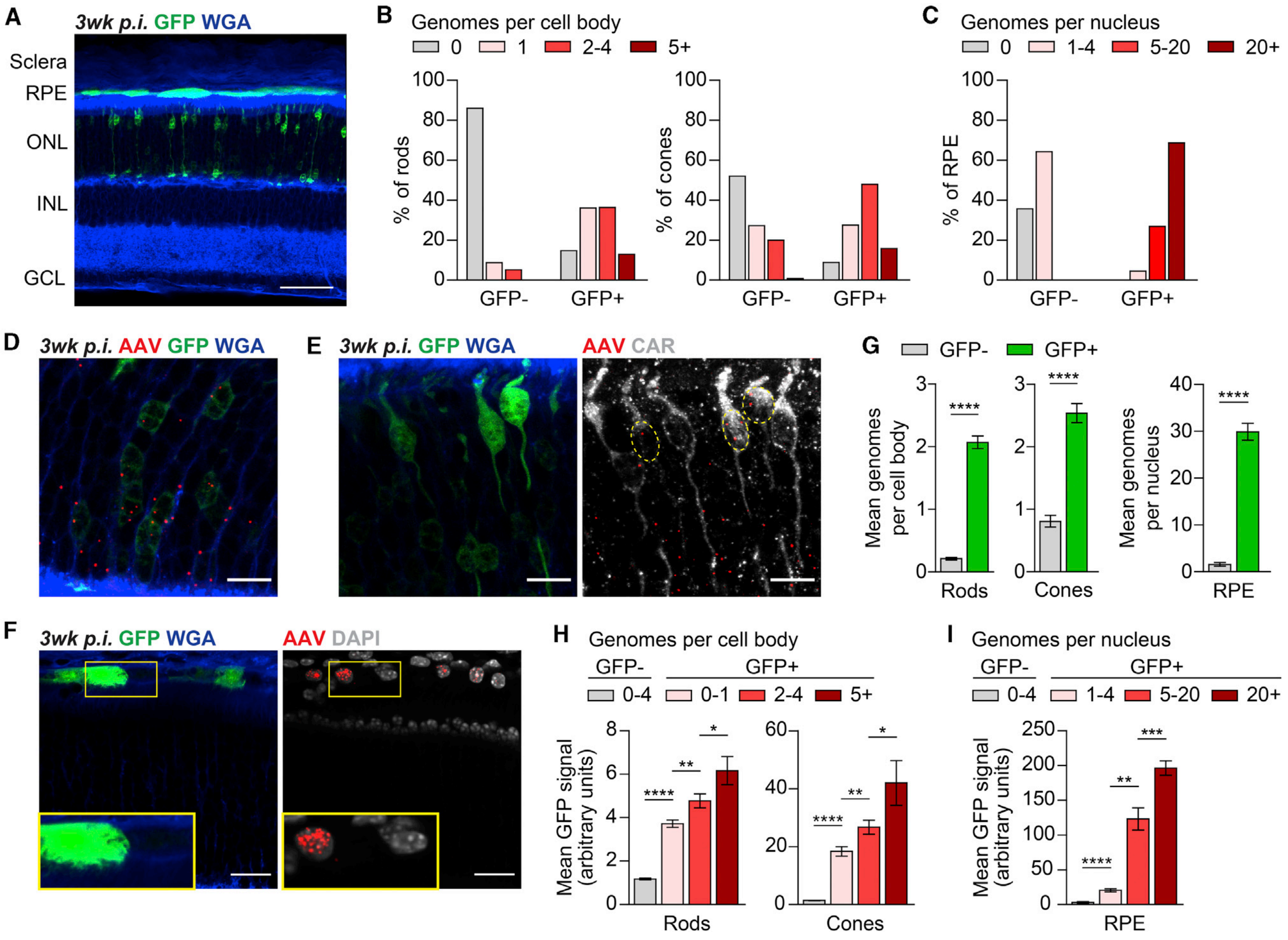

Figure 4. Comparison of AAV Genomes and GFP Expression in Photoreceptors and RPE Cells

(A) Representative section of the eye at 3 weeks after subretinal injection of $\sim 2.5 \times 10^8$ vg of AAV8-CMV-GFP demonstrating GFP expression in rods, cones, and the RPE. Scale bar, 50 μm. (B and C) Distribution of AAV genomes in the cell bodies of GFP-negative and GFP-positive photoreceptors (B) and nuclei of GFP-negative and GFP-positive RPE cells (C). In (C), there were no GFP-negative RPE cells with more than four genomes or GFP-positive RPE cells with zero genomes. (D–F) Images of rods (D), cones (E), and RPE cells (F) at 3 weeks after subretinal injection of $\sim 2.5 \times 10^8$ vg of AAV8-CMV-GFP. Rods and cones were defined as CAR-negative and CAR-positive cells in the ONL, respectively. Nuclei were labeled with DAPI. Dotted lines in (E) indicate cell bodies of GFP-positive cones. Scale bars, 10 μm for (D) and (E), 20 μm for (F). (G) Mean number of AAV genomes in the cell bodies of GFP-negative and GFP-positive photoreceptors and nuclei of GFP-negative and GFP-positive RPE cells. (H and I) Mean intensity of GFP expression in photoreceptors (H) and RPE cells (I) relative to the number of AAV genomes. n = 81–850 cell bodies or nuclei from four to eight animals per group for (B), (C), (G), (H), and (I). Data shown are mean ± SEM. *$p < 0.05$, **$p < 0.01$, ***$p < 0.001$, ****$p < 0.0001$ by two-tailed Student's t test. INL, inner nuclear layer; GCL, ganglion cell layer; CAR, cone arrestin.

photoreceptors, AAV8-CMV-GFP genomes could also be seen using SABER-FISH (Figure S3). In contrast, in regions of the retina without GFP expression, AAV8-CMV-GFP genomes could not be visualized. Collectively, our findings demonstrate that in cell types permitting promoter activity, the AAV genomes detected by SABER-FISH correlate with AAV expression.

SABER-FISH Detects AAV Genomes after Transduction of Liver and Muscle

AAV8 vectors have been used to target not only diseases of the eye, but also those of the liver and muscle.[19–22] To assess the utility of our method in other organs, SABER-FISH was thus tested in the liver and muscle of adult mice. In the liver, fluorescent puncta corresponding to AAV8-CMV-GFP genomes were absent from untreated animals but were numerous by 24 h after systemic (retro-orbital) delivery of the vector (Figure 5A). Likewise, while puncta from AAV8-CMV-GFP were not observed in untreated muscle, they could be seen in skeletal muscle nuclei within 24 h of intramuscular AAV injection (Figure 5B). These data indicate that, in addition to its applications in the retina, SABER-FISH can be used to visualize AAV genomes after transduction of liver and muscle. The diversity of these tissues further suggests that SABER-FISH may be able to track AAV genomes in a broad range of tissues for both gene therapy studies and investigations of AAV biology.

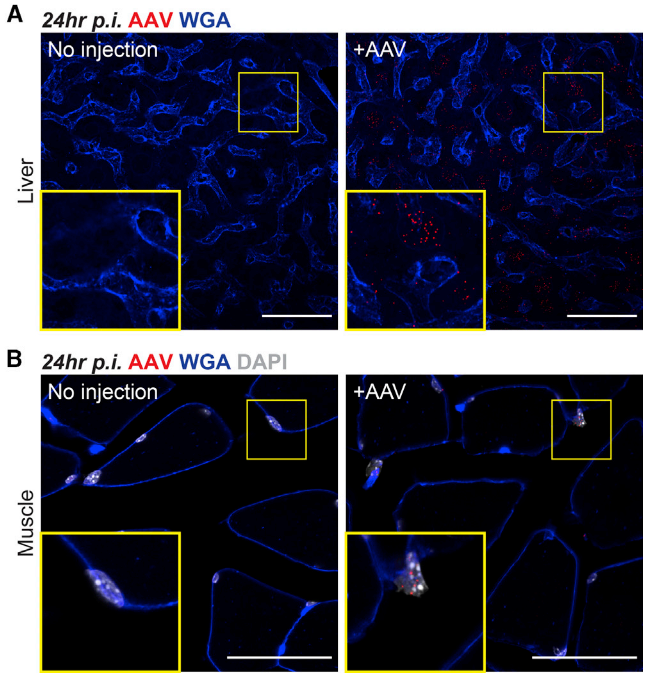

Figure 5. Detection of AAV Genomes in the Liver and Muscle

(A) Low and high magnification images of tissue from the right median lobe of the liver at 24 h after systemic injection of 5×10^{10} vg of AAV8-CMV-GFP via the retro-orbital sinus. Scale bars, 50 μm. (B) Low and high magnification images of tissue from the gastrocnemius muscle at 24 h after intramuscular injection of 5×10^{10} vg of AAV8-CMV-GFP. Scale bars, 50 μm.

AAV Vectors Can Transduce Microglia but Likely Undergo Degradation

For both basic and translational neuroscience, there is considerable interest in the development of AAV vectors that target microglia, the resident immune cells of the central nervous system. Nonetheless, few studies have observed convincing microglial expression of AAV vectors *in vivo*, leading to the conclusion that microglia are refractory to AAV transduction.[23–25] To determine whether AAV8 vectors could infect microglia in the retina, subretinal injections of AAV8-CMV-GFP were performed in CX3CR1$^{GFP/+}$ mice, in which microglia express GFP.[26] Surprisingly, AAV8-CMV-GFP genomes could be seen in nearly all CX3CR1-positive retinal microglia at 24 h p.i., indicating that these cells are efficiently transduced by AAV8 vectors (Figure 6A). Moreover, for a minority of microglia, intracellular AAV genomes were present as early as 6 h p.i. (Figure S4). To assess whether other AAV serotypes could similarly infect microglia, the same AAV genome was packaged into either AAV5, another commonly used capsid type, or Anc80, a capsid engineered to represent an ancient precursor to current serotypes.[27] With both of these vectors, AAV genomes entered cells throughout the retina (Figure S5) and could again be found in CX3CR1-positive microglia at 24 h p.i. (Figure 6B). However, unlike in other cell types, AAV genomes in retinal microglia were exclusively detected outside of nuclei (Figure 6C). These data demonstrate that AAV vectors of at least several capsid types can infect and enter microglia, but their genomes do not appear to successfully translocate into the nucleus.

To evaluate AAV expression in CX3CR1$^{GFP/+}$ animals, we subsequently used an AAV8-CMV-H2B-FusionRed vector encoding a nuclear-localized FusionRed fluorophore instead of GFP. Detection of this vector with a new set of probes (Table S4) again showed AAV genomes in most retinal microglia at 24 h p.i. (Figures 6D, 6F, and 6G), which was supported by quantitative polymerase chain reaction (qPCR) to assay for FusionRed DNA in sorted microglia (Figure S6). In contrast, at both 6 days and 3 weeks p.i., retinal microglia were devoid of AAV genomes despite their presence in adjacent cells (Figures 6D–6G), consistent with microglial degradation of AAV genomes. These observations provide a plausible explanation for the lack of AAV expression in microglia and highlight the potential of SABER-FISH to address questions pertaining to AAV vector biology.

DISCUSSION

In this study, we used SABER to amplify FISH signals from AAV genomes, allowing for the visualization of AAV vectors in the retina, liver, and muscle of mice. In the retina, these genomes were readily quantifiable and could be seen in cells as early as 3 h p.i., in agreement with prior live imaging experiments of AAV vectors in cell lines.[28] Based on a limiting dilution assay, in which a series of vector dilutions were administered *in vivo*, we determined that each fluorescent punctum detected by SABER-FISH most likely corresponded to an individual AAV genome. Within each cell, AAV genomes also appeared to localize independently, as supported by the lack of overlapping puncta in eyes that received both AAV8-CMV-GFP and AAV8-Best1-sCX3CL1. In almost all cell types examined, AAV genomes were predominantly found in nuclei at 24 h p.i., suggesting that genomes traffic to the nucleus shortly after infection. This is largely in line with previous subcellular fractionation studies of AAV vectors *in vitro*, which showed that most AAV genomes enter nuclei and specifically accumulate in nucleoli within 16 h of transduction.[29]

Following subretinal injection of AAV8-CMV-GFP, we observed a strong concordance between AAV genomes and GFP expression, particularly in rods and the RPE. Indeed, AAV8-CMV-GFP genomes were detected in ~85% of GFP-positive rods and every GFP-positive RPE cell examined in this study, implying that SABER-FISH is highly sensitive for AAV genomes in the eye. Among GFP-positive rods, many contained only one AAV genome, suggesting that if a single AAV genome makes it into a rod, it is usually sufficient to drive transgene expression. In contrast, ~48% of cones in eyes receiving AAV8-CMV-GFP were GFP-negative despite having at least one AAV genome. Whether this heterogeneity among cones was due to cell-intrinsic differences related to the CMV promoter or some uncharacterized stochastic process will be interesting to determine considering the growing number of gene therapy applications targeting photoreceptors.[30–32]

Of note, AAV genomes were especially abundant in the RPE. In fact, relative to rods and cones in the same eyes, infected RPE nuclei on

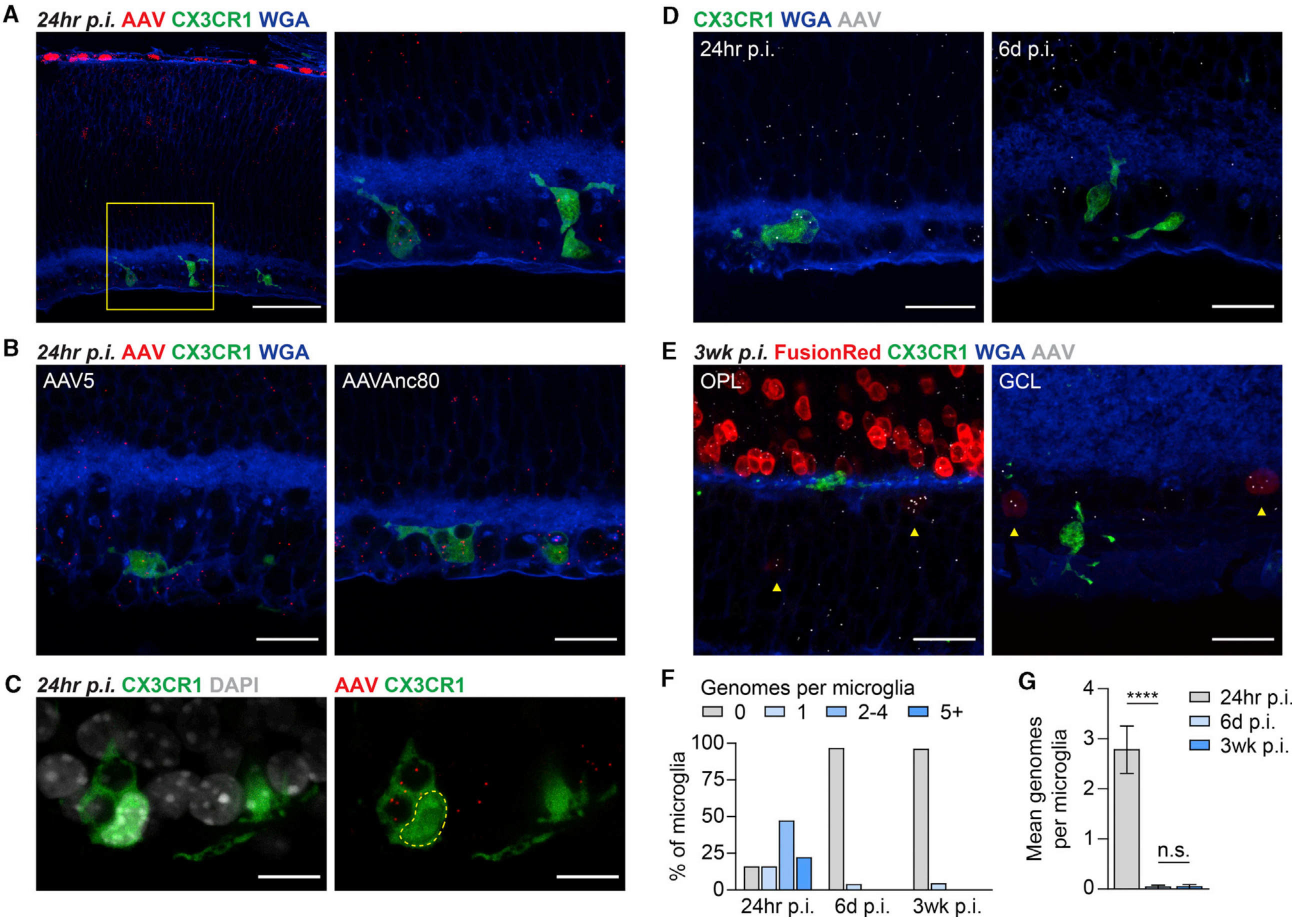

Figure 6. Detection of AAV Genomes in Retinal Microglia

(A) Low and high magnification images of AAV genomes and CX3CR1-positive microglia in CX3CR1$^{GFP/+}$ retinas at 24 h after subretinal injection of $\sim 2.5 \times 10^8$ vg of AAV8-CMV-GFP. Scale bar, 20 μm. (B) Images of AAV genomes and CX3CR1-positive microglia in CX3CR1$^{GFP/+}$ retinas at 24 h after subretinal injection of $\sim 2.5 \times 10^8$ vg of AAV5-CMV-GFP or AAVAnc80-CMV-GFP. Scale bars, 20 μm. (C) Localization of AAV8-CMV-GFP genomes relative to DAPI-labeled nuclei in CX3CR1$^{GFP/+}$ retinas at 24 h p.i. Dotted lines indicate the nucleus of a CX3CR1-positive microglia. Scale bars, 10 μm. (D and E) Images of AAV genomes, CX3CR1-positive microglia, and FusionRed expression in CX3CR1$^{GFP/+}$ retinas at 24 h (D, left), 6 days (D, right), or 3 weeks (E) after subretinal injection of $\sim 2.5 \times 10^8$ vg of AAV8-CMV-H2B-FusionRed. Retinal microglia at 3 weeks p.i. were predominantly found in the outer plexiform layer (OPL) and GCL. Arrowheads indicate cells with faint FusionRed expression. Scale bars, 20 μm. (F and G) Distribution (F) and mean number (G) of AAV genomes in CX3CR1-positive retinal microglia at 24 h or 3 weeks p.i. n = 24–32 microglia from three to five animals per group. Data shown are mean ± SEM. ****$p < 0.0001$ by two-tailed Student's t test.

average contained 10-fold the number of genomes. The high transduction rate of RPE cells may be due to a combination of their large surface area compared to photoreceptors, immediate exposure to the subretinal inoculum, and propensity to phagocytose extracellular materials.[33] In addition, AAV genomes in rods, but not cones, may have been diluted by cell divisions, as vector delivery in these experiments was performed prior to the completion of rod genesis, but after that of cones.[34] Recently, we reported that subretinally administered AAV vectors can induce cellular toxicity in mice in a dose-dependent manner.[35] This toxicity correlates with AAV expression in the RPE and consequently may be related to the accumulation of AAV genomes in these cells. However, RPE genome load alone cannot account for this phenotype, as transduction with certain AAV vectors, such as those with photoreceptor-specific promoters, does not result in toxicity.[35] Finally, even when infected with the same number of AAV genomes, cones and RPE cells in the current study exhibited much stronger GFP levels from the CMV promoter than rods. With AAV8-CMV-H2B-FusionRed, which concentrates the fluorescent signal in nuclei, we could further see faint CMV activity from genomes in retinal ganglion cells and cells of the inner nuclear layer. These results show that despite its common use as a ubiquitous promoter, CMV expression among different cell types is highly variable and occasionally difficult to detect.

It has become clear that many neurodegenerative pathologies involve genetic alterations not only in neurons, but in glial populations, such

as microglia.[36–38] The ability to directly modulate microglial gene expression with AAV vectors is therefore of interest for potential therapies addressing these disorders. Unfortunately, robust AAV expression in microglia has yet to be demonstrated *in vivo* despite the testing of numerous serotypes and promoters.[23] This has led to the belief that microglia are resistant to AAV infection and may require specially engineered capsids in order to be transduced.[23–25,39] In the present study, we found that retinal microglia are actually highly permissive to the entry of AAV vectors of at least three different serotypes, opposing the idea that expression in these cells is primarily limited by capsid entry. Rather, lack of AAV expression in microglia appears secondary to the loss of internalized AAV genomes, which are unable to reach the nucleus to initiate gene expression. While the presence of AAV genomes in microglia at 24 h p.i. could have also been from phagocytosis of infected cells, we do not favor this hypothesis, as more than 80% of microglia at this time point had genomes, meaning that nearly all microglia would have had to ingest a cell within a day of vector delivery. In some microglia, AAV genomes were even seen at 6 h p.i., further arguing against a preceding phagocytosis event. Instead, compared to the many cell types amenable to expression of AAV vectors, microglia likely possess unique machinery that allows for the recognition and destruction of intracellular viral genomes. Future investigations into the mechanisms of these processes might enable the creation of AAV vectors that can persist in microglia and drive long-term expression of transgenes.

AAV vectors in clinical trials are administered without reporter genes, such as GFP, to minimize the total dose of vectors and potential immunogenicity.[40] Identification of infected cells in patient samples consequently relies on the detection of vector transgenes, frequently by histology or qPCR. In practice, not all transgenes from AAV vectors are amenable to antibody staining or RNA FISH due to inadequate reagents, silencing of the gene, or endogenous expression in uninfected tissues. In these cases, qPCR can be used to verify successful AAV transduction, but at the expense of spatial information and the nature of the infected cell types. SABER-FISH offers a complementary assay that directly visualizes AAV genomes and identifies transduced cells in a quantitative manner. Furthermore, as demonstrated herein, the technique can be combined with SABER-FISH for RNA or immunostaining to label specific cell types, as well as SABER-FISH for other AAV vectors. We thus anticipate that SABER-FISH will be of particular value for gene therapy studies in which the locations and identities of vector-transduced cells are otherwise challenging to discern.

Finally, one potential application of SABER-FISH that should be explored is the development of an efficient method for functional AAV vector titering. Many groups, including our own, assay AAV vectors with qPCR to adjust the concentration of different stocks and ensure that comparisons among vectors are fair and reproducible.[41] While this approach in principle corrects for variations in the number of AAV genomes per unit volume, it has been shown to be highly inconsistent across laboratories, even when measuring the same stock.[42] Moreover, qPCR of packaged AAV DNA cannot account for how many of these genomes are capable of carrying out productive infections. Functional titering, which involves transducing cells with a given vector and measuring some output of transgene expression, can instead be used to determine the infectivity of vector stocks.[43] However, the process is often time-consuming, since transgenes may take several days to become detectable, and it requires the promoter of the vector to be expressed in the tested cells. As shown in this study, SABER-FISH can visualize and quantify intracellular AAV genomes within hours of vector delivery, bypassing the major limitations of current titering methods. An optimized SABER-FISH protocol for functional AAV vector titering may therefore further facilitate the dissemination of AAV-based therapies.

MATERIALS AND METHODS

Animals

All experiments were approved by the Institutional Animal Care and Use Committee (IACUC) of Harvard University and performed in accordance with institutional guidelines. CD-1 (#022) animals were purchased from Charles River Laboratories. CX3CR1$^{GFP/GFP}$ (#005582) mice were purchased from The Jackson Laboratory and crossed with CD-1 mice to obtain CX3CR1$^{GFP/+}$ animals. Mice were subsequently maintained at Harvard Medical School on a 12-hour alternating light and dark cycle.

AAV Vector Production

All AAV vectors used in this study were single-stranded. The AAV8-CMV-GFP plasmid was obtained from the Harvard DF/HCC DNA Resource Core (clone ID: EvNO00061595) and consisted of a CMV promoter, β-globin intron, cDNA sequence for GFP, and β-globin polyadenylation sequence. The AAV8-Best1-sCX3CL1 plasmid was synthesized as previously reported and consisted of a Best1 promoter, SV40 intron, cDNA sequence for soluble CX3CL1, woodchuck hepatitis virus posttranscriptional regulatory element, and bovine growth hormone polyadenylation sequence.[17] The AAV8-CMV-H2B-FusionRed plasmid was synthesized by replacing the GFP sequence of the AAV8-CMV-GFP plasmid with the H2B-FusionRed sequence of pFusionRed-H2B (Evrogen) using Gibson assembly. Recombinant AAV8 vectors were generated as previously described by using polyethylenimine to transfect HEK293T cells with a mixture of the AAV plasmid, adenovirus helper plasmid, and rep2/cap8 packaging plasmid.[44] AAV5-CMV-GFP and AAVAnc80-CMV-GFP vectors were generated in an identical manner by transfecting with the AAV8-CMV-GFP plasmid, adenovirus helper plasmid, and rep2/cap5 or Anc80L65 (a gift from L. Vandenberghe, Harvard Medical School) packaging plasmid.[27] Supernatant was collected 72 h after transfection, and viral particles were PEGylated overnight and precipitated by centrifugation. Viral particles were then centrifuged through an iodixanol gradient to remove cellular debris, and the recovered vector was washed three times with phosphate-buffered saline (PBS) and collected in a final volume of 100–200 μL. Purified AAV vectors were semiquantitatively titered by SYPRO Ruby (Molecular Probes) staining for viral capsid proteins (VP1, VP2, and VP3) in comparison to a reference vector titered by qPCR.

Probe Design and Synthesis

SABER-FISH probes targeting the genomes of AAV8-CMV-GFP, AAV8-Best1-sCX3CL1, and AAV8-CMV-H2B-FusionRed were designed *in silico* using ApE software. All probes were generated to recognize only the negative-sense strand of the vector. For each vector sequence, we first identified a list of non-overlapping oligonucleotides that were (1) complementary to the genome, (2) at least 36 nt long, and (3) predicted to have a melting temperature between 67°C and 71°C based on ApE's default parameters. A 9-nt primer (5′-CATCATCAT-3′ or 5′-CAACTTAAC-3′) with a TTT linker was appended to the 3′ ends of these oligonucleotides to finalize the probe sequences, which were then ordered from Integrated DNA Technologies with standard desalting in a 96-well plate at the 10 nM synthesis scale. Probes were pooled into a single tube with a multi-channel pipette and extended to ~500 nt using a previously described primer exchange reaction, in which a catalytic hairpin (Table S5) and strand-displacing polymerase repeatedly added the same sequence to the end of the 9-nt primer.[13] Extended probe sets were subsequently purified with MinElute PCR purification columns (QIAGEN) and eluted in nuclease-free water. Probes targeting *Grik1* were designed as previously reported with sequences listed in Kishi et al.[13] Sequences for all other probes used in this study can be found in Tables S1–S4.

AAV Vector Delivery

Subretinal injections were performed on neonatal mice (postnatal day 0–1) as previously described unless otherwise noted.[17,35] After anesthetizing the animal on ice, the palpebral fissure was carefully opened with a 30G needle and the eye was exposed. Using a glass needle controlled by a FemtoJet microinjector (Eppendorf), ~0.25 μL of AAV vectors (1×10^{11} to 1×10^{12} vector genomes [vg]/mL) or purified plasmid (1 mg/mL) was then injected into the subretinal space. Both left and right eyes were used for injections. Transduction of liver and muscle was performed on 6- to 10-week-old male CD-1 mice. For liver, 100 μL of vector (5×10^{11} vg/mL) was injected into the retro-orbital sinus. For muscle, 25 μL of vector (2×10^{12} vg/mL) was injected intramuscularly into the gastrocnemius of the right hind leg.

Tissue Preparation

Freshly enucleated eyes were immediately dissected in PBS to remove the cornea, iris, ciliary body, and lens. The remaining eye cup was fixed in 4% paraformaldehyde (PFA) for 1 h at room temperature, cryoprotected in a sucrose gradient, and embedded in a 1:1 solution of 30% sucrose in PBS and optimal cutting temperature (OCT) compound (Tissue-Tek) frozen on dry ice. For liver studies, mice anesthetized with ketamine and xylazine were intracardially perfused with 4% PFA and a portion of the right median lobe excised and immersed in 4% PFA overnight at 4°C. For muscle studies, the gastrocnemius muscle of the right hind leg was excised, cut in half, and immersed in 4% PFA overnight at 4°C. Fixed liver and muscle tissues were subsequently cryoprotected and embedded as detailed above for fixed eye cups. Embedded tissues were cut on a Leica CM3050S cryostat (Leica Microsystems) into 30-μm sections for eye cups and 20-μm sections for liver and muscle. For retinal flat-mounts, retinas were carefully dissected from the rest of the eye and fixed in 4% PFA for 30 min at room temperature. After PBS washes, retinas were relaxed with four radial incisions with the ganglion cell layer facing up, flattened onto a microscope slide, and mounted using Fluoromount-G (SouthernBiotech).

SABER-FISH

Detection of AAV genomes and cell-type mRNAs by SABER-FISH was performed in 350-μL chamber wells (Grace Bio-Labs) using the RNA ISH protocol from Kishi et al.[13] with minor modifications. Briefly, tissues were sectioned onto Superfrost Plus Micro slides (VWR) pretreated with a 0.3 mg/mL solution of poly-D-lysine in 2× borate buffer for 30 min at room temperature. After rehydration with PBSTw (PBS + 0.1% Tween 20), samples were washed with a wash-hybridization solution (40% formamide + 1% Tween 20 + 2× saline sodium citrate [SSC]) at 43°C followed by incubation in hybridization solution (40% formamide + 10% dextran sulfate + 1% Tween 20 + 2× SSC) containing 8.33 μg/mL of each probe set for 16 h at 43°C. Samples subsequently underwent two 30-min washes with wash-hybridization solution at 43°C and two 5-min washes with PBSTw at room temperature. Hybridized probes were then detected by incubating with 0.2 μM fluorescent oligonucleotides (IDT, Table S5) in PBS + 0.2% Tween 20 for 30 min at 37°C. Cell boundaries were visualized by staining with 10 μg/mL WGA (Biotium) in PBSTw for 2 h at room temperature. Nuclei were labeled by staining with 0.5 μg/mL DAPI (Invitrogen) in PBS for 5 min at room temperature. When applicable, samples were treated with 100 μg/mL RNase A (Thermo Scientific) in PBSTw for 30 min at 37°C prior to the first use of wash-hybridization solution. Slides were mounted with an 80% glycerol medium containing 1× PBS, 20 mM Tris (pH 8.0), and 2.5 mg/mL propyl gallate.

Immunostaining

After FISH and labeling with WGA, tissues were treated with blocking solution (PBS + 5% goat serum + 0.1% Triton X-100) for 1 h at room temperature followed by incubation with 1:3,000 of rabbit anti-mouse cone arrestin (Millipore, AB15282) in blocking solution for 16 h at 4°C. Tissues were subsequently washed with PBS and incubated with 1:1,000 of donkey anti-rabbit Alexa Fluor 647 secondary antibody (Jackson ImmunoResearch, 711-605-152) in PBS for 2 h at room temperature prior to slide mounting.

Image Acquisition and Analysis

Imaging was conducted on Zeiss LSM 710 and LSM 780 single-point scanning confocal microscopes using ×20 air, ×40 oil, and ×63 oil objectives. Laser lines were 405, 488, 561, and 633 nm. FISH images were acquired as z stacks, processed with ImageJ, and displayed as maximum-intensity projections of equal depth for images within the same panel. For all images of the eye, the RPE-facing side of the retina was oriented toward the top. Automated cell segmentation and puncta detection were performed as previously described with minor modifications.[13] For retinal samples, cells in 3D z stacks were first segmented based on WGA labeling of cell membranes using

ACME, an open-source segmentation software.[16] Puncta were then detected and quantified with PD3D (https://github.com/ewest11/PD3D), a MATLAB script using a Laplacian of Gaussian method to distinguish puncta from tissue background and assign them to segmented cell bodies. Cells adjoining any boundary of the 3D image or located outside the neural retina were computationally removed to confine analyses of puncta to whole retinal cells. For RPE and microglia, puncta were again detected using PD3D but manually assigned to RPE nuclei or CX3CR1-positive microglia. Quantification of colocalized puncta in 3D images were performed using coloc3D (https://mathworks.com/matlabcentral/fileexchange/5694-coloc3d), a MATLAB script identifying overlapping particles in z stack images. For each 3D image, the fraction of red puncta that colocalized with green and green puncta that colocalized with red was determined, and these two values were averaged to calculate the probability of colocalization. Measurements of GFP expression were manually performed in ImageJ by drawing a mask around each cell body for photoreceptors or nucleus for RPE and recording the mean GFP intensity.

Statistical Analysis

Statistical analyses were performed using GraphPad Prism software. For all statistical tests, a p value less than 0.05 was considered significant.

SUPPLEMENTAL INFORMATION

Supplemental Information can be found online at https://doi.org/10.1016/j.omtm.2020.10.003.

AUTHOR CONTRIBUTIONS

S.K.W., S.W.L., and C.L.C. designed the study. S.K.W., S.W.L., C.M.H., and T.B.K. performed the experiments and analyzed the data. S.K.W. and C.L.C. wrote the manuscript with input from all authors.

CONFLICTS OF INTEREST

The authors declare no competing interests.

ACKNOWLEDGMENTS

The authors thank Emma West, Sophia Zhao, Xiang Ma, Ryan Delgado, Paula Montero-Llopis, Microscopy Resources on the North Quad at Harvard Medical School, and the Brigham and Women's Hospital Flow Cytometry Core for discussions and technical support. The graphical abstract was created with BioRender. This work was supported by the Howard Hughes Medical Institute (to S.K.W. and C.L.C.) and the Foundation Fighting Blindness (to S.K.W.).

REFERENCES

1. Hermonat, P.L., and Muzyczka, N. (1984). Use of adeno-associated virus as a mammalian DNA cloning vector: transduction of neomycin resistance into mammalian tissue culture cells. Proc. Natl. Acad. Sci. USA *81*, 6466–6470.
2. Tratschin, J.D., West, M.H., Sandbank, T., and Carter, B.J. (1984). A human parvovirus, adeno-associated virus, as a eucaryotic vector: transient expression and encapsidation of the procaryotic gene for chloramphenicol acetyltransferase. Mol. Cell. Biol. *4*, 2072–2081.
3. Daya, S., and Berns, K.I. (2008). Gene therapy using adeno-associated virus vectors. Clin. Microbiol. Rev. *21*, 583–593.
4. Sehara, Y., Fujimoto, K.I., Ikeguchi, K., Katakai, Y., Ono, F., Takino, N., Ito, M., Ozawa, K., and Muramatsu, S.I. (2017). Persistent expression of dopamine-synthesizing enzymes 15 years after gene transfer in a primate model of Parkinson's disease. Hum. Gene Ther. Clin. Dev. *28*, 74–79.
5. Srivastava, A., Lusby, E.W., and Berns, K.I. (1983). Nucleotide sequence and organization of the adeno-associated virus 2 genome. J. Virol. *45*, 555–564.
6. Smith, R.H. (2008). Adeno-associated virus integration: virus versus vector. Gene Ther. *15*, 817–822.
7. Penaud-Budloo, M., Le Guiner, C., Nowrouzi, A., Toromanoff, A., Chérel, Y., Chenuaud, P., Schmidt, M., von Kalle, C., Rolling, F., Moullier, P., and Snyder, R.O. (2008). Adeno-associated virus vector genomes persist as episomal chromatin in primate muscle. J. Virol. *82*, 7875–7885.
8. Maguire, A.M., Simonelli, F., Pierce, E.A., Pugh, E.N., Jr., Mingozzi, F., Bennicelli, J., Banfi, S., Marshall, K.A., Testa, F., Surace, E.M., et al. (2008). Safety and efficacy of gene transfer for Leber's congenital amaurosis. N. Engl. J. Med. *358*, 2240–2248.
9. Mendell, J.R., Al-Zaidy, S., Shell, R., Arnold, W.D., Rodino-Klapac, L.R., Prior, T.W., Lowes, L., Alfano, L., Berry, K., Church, K., et al. (2017). Single-dose gene-replacement therapy for spinal muscular atrophy. N. Engl. J. Med. *377*, 1713–1722.
10. Kothari, P., De, B.P., He, B., Chen, A., Chiuchiolo, M.J., Kim, D., Nikolopoulou, A., Amor-Coarasa, A., Dyke, J.P., Voss, H.U., et al. (2017). Radioiodinated capsids facilitate in vivo non-invasive tracking of adeno-associated gene transfer vectors. Sci. Rep. *7*, 39594.
11. Kelich, J.M., Ma, J., Dong, B., Wang, Q., Chin, M., Magura, C.M., Xiao, W., and Yang, W. (2015). Super-resolution imaging of nuclear import of adeno-associated virus in live cells. Mol. Ther. Methods Clin. Dev. *2*, 15047.
12. Lux, K., Goerlitz, N., Schlemminger, S., Perabo, L., Goldnau, D., Endell, J., Leike, K., Kofler, D.M., Finke, S., Hallek, M., and Büning, H. (2005). Green fluorescent protein-tagged adeno-associated virus particles allow the study of cytosolic and nuclear trafficking. J. Virol. *79*, 11776–11787.
13. Kishi, J.Y., Lapan, S.W., Beliveau, B.J., West, E.R., Zhu, A., Sasaki, H.M., Saka, S.K., Wang, Y., Cepko, C.L., and Yin, P. (2019). SABER amplifies FISH: enhanced multiplexed imaging of RNA and DNA in cells and tissues. Nat. Methods *16*, 533–544.
14. Sarra, G.M., Stephens, C., Schlichtenbrede, F.C., Bainbridge, J.W.B., Thrasher, A.J., Luthert, P.J., and Ali, R.R. (2002). Kinetics of transgene expression in mouse retina following sub-retinal injection of recombinant adeno-associated virus. Vision Res. *42*, 541–549.
15. Natkunarajah, M., Trittibach, P., McIntosh, J., Duran, Y., Barker, S.E., Smith, A.J., Nathwani, A.C., and Ali, R.R. (2008). Assessment of ocular transduction using single-stranded and self-complementary recombinant adeno-associated virus serotype 2/8. Gene Ther. *15*, 463–467.
16. Mosaliganti, K.R., Noche, R.R., Xiong, F., Swinburne, I.A., and Megason, S.G. (2012). ACME: automated cell morphology extractor for comprehensive reconstruction of cell membranes. PLoS Comput. Biol. *8*, e1002780.
17. Wang, S.K., Xue, Y., Rana, P., Hong, C.M., and Cepko, C.L. (2019). Soluble CX3CL1 gene therapy improves cone survival and function in mouse models of retinitis pigmentosa. Proc. Natl. Acad. Sci. USA *116*, 10140–10149.
18. Chang, B., Hawes, N.L., Hurd, R.E., Davisson, M.T., Nusinowitz, S., and Heckenlively, J.R. (2002). Retinal degeneration mutants in the mouse. Vision Res. *42*, 517–525.
19. Nathwani, A.C., Tuddenham, E.G.D., Rangarajan, S., Rosales, C., McIntosh, J., Linch, D.C., Chowdary, P., Riddell, A., Pie, A.J., Harrington, C., et al. (2011). Adenovirus-associated virus vector-mediated gene transfer in hemophilia B. N. Engl. J. Med. *365*, 2357–2365.
20. Mack, D.L., Poulard, K., Goddard, M.A., Latournerie, V., Snyder, J.M., Grange, R.W., Elverman, M.R., Denard, J., Veron, P., Buscara, L., et al. (2017). Systemic AAV8-mediated gene therapy drives whole-body correction of myotubular myopathy in dogs. Mol. Ther. *25*, 839–854.
21. Childers, M.K., Joubert, R., Poulard, K., Moal, C., Grange, R.W., Doering, J.A., Lawlor, M.W., Rider, B.E., Jamet, T., Danièle, N., et al. (2014). Gene therapy prolongs survival and restores function in murine and canine models of myotubular myopathy. Sci. Transl. Med. *6*, 220ra10.

22. Sabatino, D.E., Lange, A.M., Altynova, E.S., Sarkar, R., Zhou, S., Merricks, E.P., Franck, H.G., Nichols, T.C., Arruda, V.R., and Kazazian, H.H., Jr. (2011). Efficacy and safety of long-term prophylaxis in severe hemophilia A dogs following liver gene therapy using AAV vectors. Mol. Ther. *19*, 442–449.

23. Maes, M.E., Colombo, G., Schulz, R., and Siegert, S. (2019). Targeting microglia with lentivirus and AAV: recent advances and remaining challenges. Neurosci. Lett. *707*, 134310.

24. Chakrabarty, P., Rosario, A., Cruz, P., Siemienski, Z., Ceballos-Diaz, C., Crosby, K., Jansen, K., Borchelt, D.R., Kim, J.Y., Jankowsky, J.L., et al. (2013). Capsid serotype and timing of injection determines AAV transduction in the neonatal mice brain. PLoS ONE *8*, e67680.

25. Kanaan, N.M., Sellnow, R.C., Boye, S.L., Coberly, B., Bennett, A., Agbandje-McKenna, M., Sortwell, C.E., Hauswirth, W.W., Boye, S.E., and Manfredsson, F.P. (2017). Rationally engineered AAV capsids improve transduction and volumetric spread in the CNS. Mol. Ther. Nucleic Acids *8*, 184–197.

26. Jung, S., Aliberti, J., Graemmel, P., Sunshine, M.J., Kreutzberg, G.W., Sher, A., and Littman, D.R. (2000). Analysis of fractalkine receptor CX_3CR1 function by targeted deletion and green fluorescent protein reporter gene insertion. Mol. Cell. Biol. *20*, 4106–4114.

27. Zinn, E., Pacouret, S., Khaychuk, V., Turunen, H.T., Carvalho, L.S., Andres-Mateos, E., Shah, S., Shelke, R., Maurer, A.C., Plovie, E., et al. (2015). In silico reconstruction of the viral evolutionary lineage yields a potent gene therapy vector. Cell Rep. *12*, 1056–1068.

28. Cervelli, T., Palacios, J.A., Zentilin, L., Mano, M., Schwartz, R.A., Weitzman, M.D., and Giacca, M. (2008). Processing of recombinant AAV genomes occurs in specific nuclear structures that overlap with foci of DNA-damage-response proteins. J. Cell Sci. *121*, 349–357.

29. Johnson, J.S., and Samulski, R.J. (2009). Enhancement of adeno-associated virus infection by mobilizing capsids into and out of the nucleolus. J. Virol. *83*, 2632–2644.

30. Rakoczy, E.P., Lai, C.M., Magno, A.L., Wikstrom, M.E., French, M.A., Pierce, C.M., Schwartz, S.D., Blumenkranz, M.S., Chalberg, T.W., Degli-Esposti, M.A., and Constable, I.J. (2015). Gene therapy with recombinant adeno-associated vectors for neovascular age-related macular degeneration: 1 year follow-up of a phase 1 randomised clinical trial. Lancet *386*, 2395–2403.

31. Pichard, V., Provost, N., Mendes-Madeira, A., Libeau, L., Hulin, P., Tshilenge, K.T., Biget, M., Ameline, B., Deschamps, J.Y., Weber, M., et al. (2016). AAV-mediated gene therapy halts retinal degeneration in PDE6β-deficient dogs. Mol. Ther. *24*, 867–876.

32. Banin, E., Gootwine, E., Obolensky, A., Ezra-Elia, R., Ejzenberg, A., Zelinger, L., Honig, H., Rosov, A., Yamin, E., Sharon, D., et al. (2015). Gene augmentation therapy restores retinal function and visual behavior in a sheep model of *CNGA3* achromatopsia. Mol. Ther. *23*, 1423–1433.

33. Young, R.W., and Bok, D. (1969). Participation of the retinal pigment epithelium in the rod outer segment renewal process. J. Cell Biol. *42*, 392–403.

34. Cepko, C. (2014). Intrinsically different retinal progenitor cells produce specific types of progeny. Nat. Rev. Neurosci. *15*, 615–627.

35. Xiong, W., Wu, D.M., Xue, Y., Wang, S.K., Chung, M.J., Ji, X., Rana, P., Zhao, S.R., Mai, S., and Cepko, C.L. (2019). AAV *cis*-regulatory sequences are correlated with ocular toxicity. Proc. Natl. Acad. Sci. USA *116*, 5785–5794.

36. Mathys, H., Adaikkan, C., Gao, F., Young, J.Z., Manet, E., Hemberg, M., De Jager, P.L., Ransohoff, R.M., Regev, A., and Tsai, L.H. (2017). Temporal tracking of microglia activation in neurodegeneration at single-cell resolution. Cell Rep. *21*, 366–380.

37. Hammond, T.R., Dufort, C., Dissing-Olesen, L., Giera, S., Young, A., Wysoker, A., Walker, A.J., Gergits, F., Segel, M., Nemesh, J., et al. (2019). Single-cell RNA sequencing of microglia throughout the mouse lifespan and in the injured brain reveals complex cell-state changes. Immunity *50*, 253–271.e6.

38. Wang, S.K., Xue, Y., and Cepko, C.L. (2020). Microglia modulation by TGF-β1 protects cones in mouse models of retinal degeneration. J. Clin. Invest. *130*, 4360–4369.

39. Rosario, A.M., Cruz, P.E., Ceballos-Diaz, C., Strickland, M.R., Siemienski, Z., Pardo, M., Schob, K.L., Li, A., Aslanidi, G.V., Srivastava, A., et al. (2016). Microglia-specific targeting by novel capsid-modified AAV6 vectors. Mol. Ther. Methods Clin. Dev. *3*, 16026.

40. Gao, G., Wang, Q., Calcedo, R., Mays, L., Bell, P., Wang, L., Vandenberghe, L.H., Grant, R., Sanmiguel, J., Furth, E.E., and Wilson, J.M. (2009). Adeno-associated virus-mediated gene transfer to nonhuman primate liver can elicit destructive transgene-specific T cell responses. Hum. Gene Ther. *20*, 930–942.

41. Veldwijk, M.R., Topaly, J., Laufs, S., Hengge, U.R., Wenz, F., Zeller, W.J., and Fruehauf, S. (2002). Development and optimization of a real-time quantitative PCR-based method for the titration of AAV-2 vector stocks. Mol. Ther. *6*, 272–278.

42. Ayuso, E., Blouin, V., Lock, M., McGorray, S., Leon, X., Alvira, M.R., Auricchio, A., Bucher, S., Chtarto, A., Clark, K.R., et al. (2014). Manufacturing and characterization of a recombinant adeno-associated virus type 8 reference standard material. Hum. Gene Ther. *25*, 977–987.

43. Mohiuddin, I., Loiler, S., Zolotukhin, I., Byrne, B.J., Flotte, T.R., and Snyder, R.O. (2005). Herpesvirus-based infectious titering of recombinant adeno-associated viral vectors. Mol. Ther. *11*, 320–326.

44. Grieger, J.C., Choi, V.W., and Samulski, R.J. (2006). Production and characterization of adeno-associated viral vectors. Nat. Protoc. *1*, 1412–1428.

OMTM, Volume *19*

Supplemental Information

In Situ Detection of Adeno-associated Viral Vector Genomes with SABER-FISH

Sean K. Wang, Sylvain W. Lapan, Christin M. Hong, Tyler B. Krause, and Constance L. Cepko

SUPPLEMENTAL METHODS

Cell sorting. Microglia from CX3CR1$^{GFP/+}$ mouse retinas were isolated using a BD FACSAria Fusion as previously described with minor modifications.[1] For each sample, four freshly dissected retinas were pooled and dissociated using cysteine-activated papain followed by gentle trituration with a micropipette. Samples were subsequently washed with FACS buffer (PBS containing 2% fetal bovine serum and 2mM ethylenediaminetetraacetic acid [EDTA]), passed through a 40 µm filter, and stained with 0.5 µg/mL of DAPI (Invitrogen) to exclude non-viable cells. Analysis of sorted populations was performed using FlowJo 10 (Tree Star).

qPCR. Total DNA including viral DNA was purified from sorted CX3CR1-positive microglia using a *Quick*-DNA Microprep Plus Kit (Zymo Research). Total RNA was purified from whole retinas (pre-sort) or sorted CX3CR1-positive microglia (post-sort) using an RNeasy Micro Kit (Qiagen). For RNA samples, cDNA was synthesized using the SuperScript III First-Strand Synthesis System (Invitrogen) with oligo(dT) primers. Total DNA or cDNA templates were then assayed by qPCR using the Power SYBR Green PCR Master Mix (Applied Biosystems) on a CFX96 real-time PCR detection system (BioRad). Reactions were performed in duplicate with the primer sequences listed in Table S6. RNA expression was normalized to that of the housekeeping gene *Gapdh*. FusionRed DNA copy number was normalized to that of genomic Gapdh.

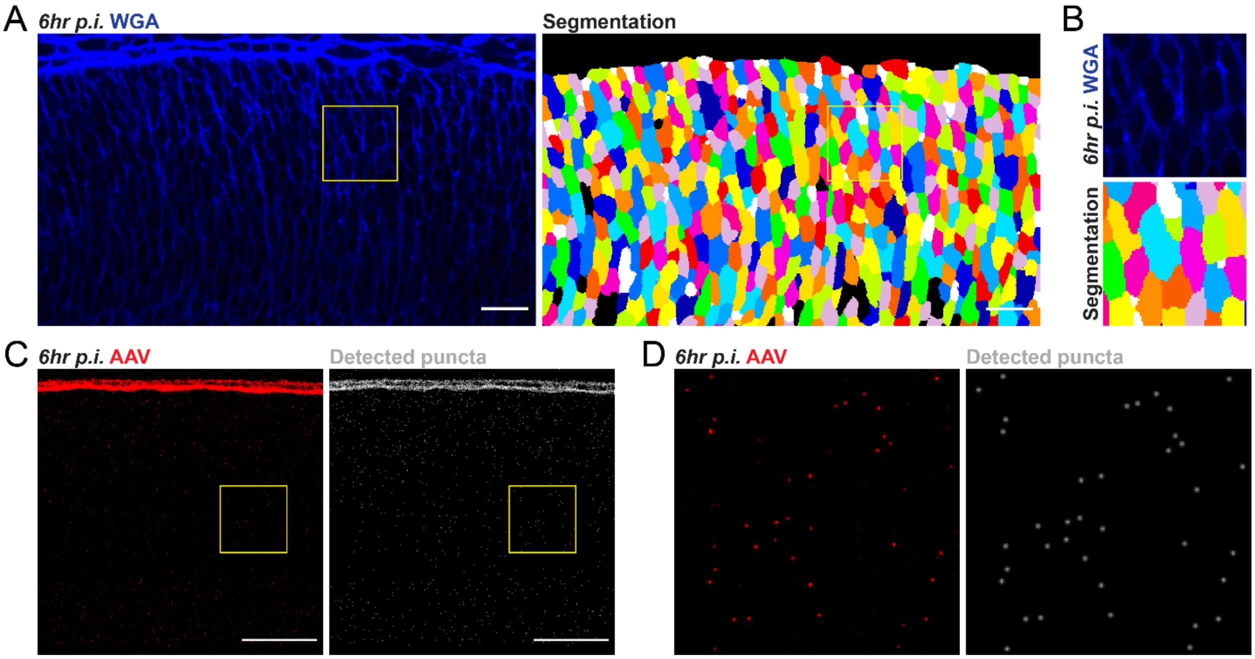

Figure S1. Automated cell segmentation and puncta detection.

(**A**, **B**) Low (A) and high (B) magnification images of cell membranes in the retina visualized with WGA and the corresponding output following automated cell segmentation.[2] Different colors indicate boundaries of adjoining cell bodies. Scale bars, 20 µm. (**C**, **D**) Low (C) and high (D) magnification images of fluorescent puncta at 6 hours after subretinal injection of ~2.5 x 10^8 vg of AAV8-CMV-GFP and the corresponding puncta following automated puncta detection.[3] Scale bars, 50 µm.

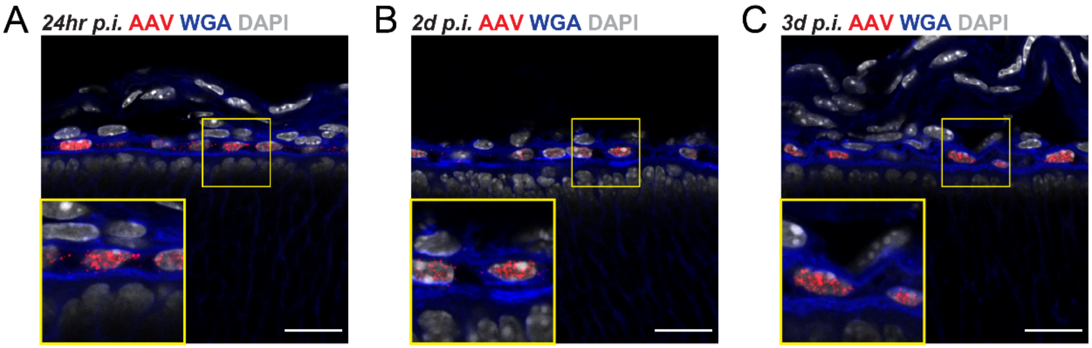

Figure S2. Nuclear localization of AAV genomes in the RPE.

(**A-C**) Low and high magnification images of AAV genomes in RPE nuclei at 24 hours (A), two days (B), and three days (C) after subretinal injection of ~2.5 x 10^8 vg of AAV8-CMV-GFP. Nuclei were labeled with DAPI. Scale bars, 20 µm.

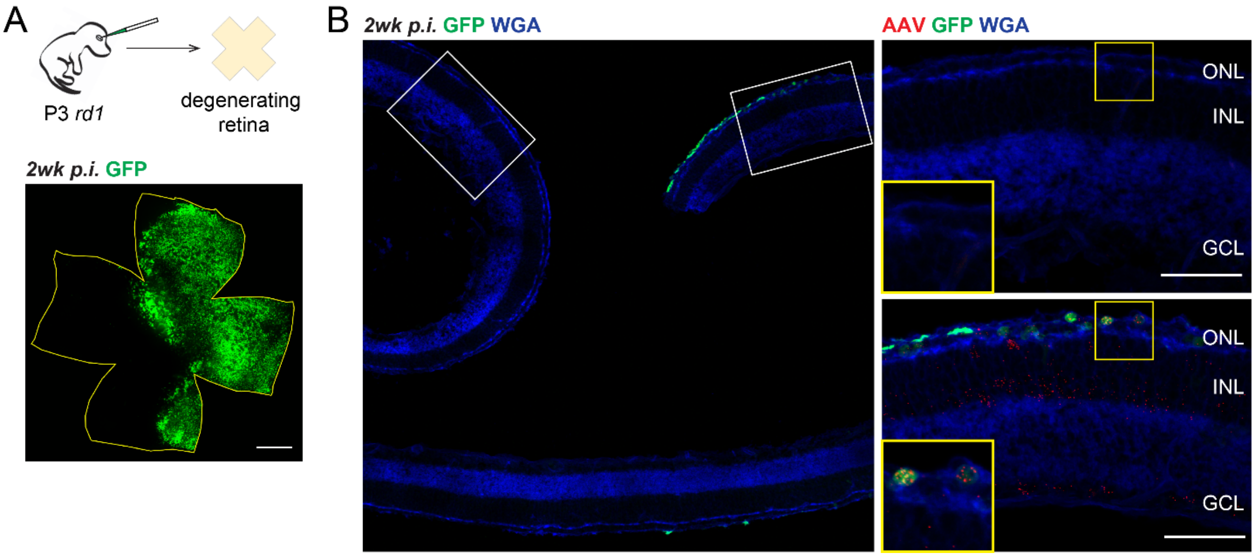

Figure S3. AAV genomes in the *rd1* disease model.

(**A**) Partial infection of a degenerating *rd1* retina after subretinal injection of ~2.5 x 10^8 vg of AAV8-CMV-GFP on postnatal day 3. Boundaries of the flat-mounted retina are outlined in yellow. Scale bar, 1 mm. (**B**) Low and high magnification images of AAV8-CMV-GFP genomes in GFP-negative (top) and GFP-positive (bottom) regions of the retina. Only a thin layer of photoreceptors remains in the ONL at this time point due to severe retinal degeneration. Scale bars, 50 μm. INL, inner nuclear layer; GCL, ganglion cell layer.

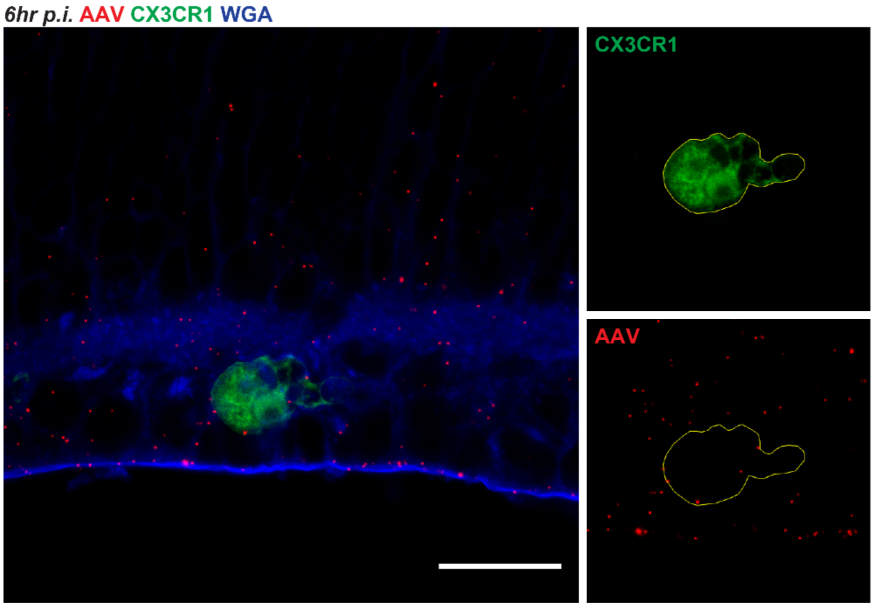

Figure S4. AAV genomes in retinal microglia at 6 hours post-injection.
Example of a CX3CR1-positive microglia with intracellular AAV genomes at 6 hours after subretinal injection of ~2.5 x 10^8 vg of AAV8-CMV-GFP. Cell boundaries of the microglia are outlined in yellow. Scale bar, 20 µm.

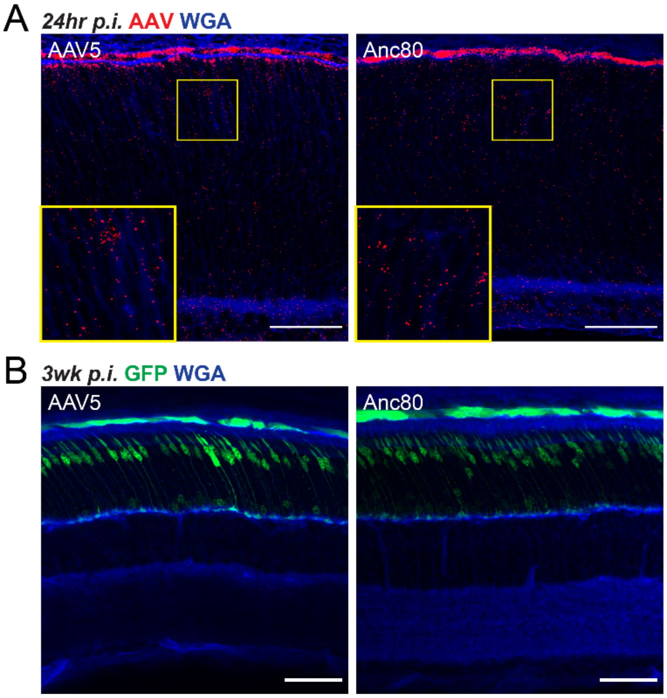

Figure S5. Distribution of AAV5-CMV-GFP and AAVAnc80-CMV-GFP in the retina.

(**A**) Low and high magnification images of retinas at 24 hours after subretinal injection of ~2.5 x 10^8 vg of AAV5-CMV-GFP or AAVAnc80-CMV-GFP. Scale bars, 50 µm. (**B**) Representative sections of the eye at 3 weeks after subretinal injection of ~2.5 x 10^8 vg of AAV5-CMV-GFP or AAVAnc80-CMV-GFP. Both vectors resulted in GFP expression in rods, cones, and the RPE. Scale bars, 50 µm.

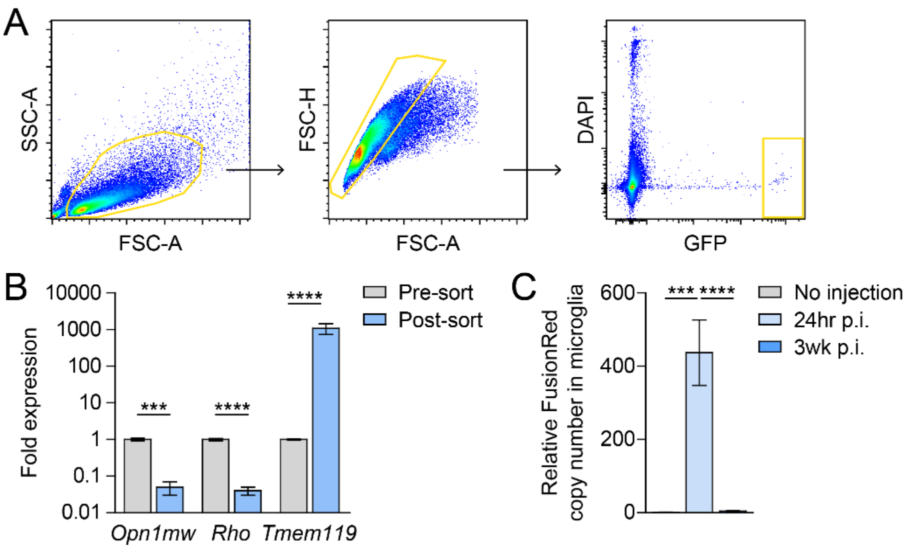

Figure S6. Detection of AAV genomes in retinal microglia by qPCR.

(**A**) Representative fluorescence-activated cell sorting (FACS) gating to isolate GFP-positive microglia from CX3CR1$^{GFP/+}$ retinas. (**B**) RNA expression of cone- (*Opn1mw*), rod- (*Rho*), and microglia- (*Tmem119*)[4] specific genes before and after sorting from three-week-old CX3CR1$^{GFP/+}$ retinas. Fold changes are relative to unsorted cells. (**C**) Copy number of FusionRed in sorted microglia at 24 hours or 3 weeks after subretinal injection of ~2.5 x 10^8 vg of AAV8-CMV-H2B-FusionRed. Values are relative to microglia from uninjected postnatal day 1 retinas. n = 3-4 animals per group for (B) and (C). Data shown are mean ± SEM. *** P<0.001, **** P<0.0001 by two-tailed Student's t-test.

Table S1. SABER-FISH probe sequences for AAV8-CMV-GFP.

ID	*Sequence*
CMVGFP_27_1	CAGAGAGGGAGTGGCCAACTCCATCACTAGGGGTTCTTTCATCATCAT
CMVGFP_27_2	AGTTAATGATTAACCCGCCATGCTACTTATCTACGTAGCCATTTCATCATCAT
CMVGFP_27_3	AAAATTAGTCAGCCATGAGCTTGGCCCATTGCATACTTTCATCATCAT
CMVGFP_27_4	TGTACATTTATATTGGCTCATGTCCAACATTACCGCCATGTTTTCATCATCAT
CMVGFP_27_5	ATTACGGGGTCATTAGTTCATAGCCCATATATGGAGTTCCGTTTCATCATCAT
CMVGFP_27_6	CGTTACATAACTTACGGTAAATGGCCCGCCTGGCTGTTTCATCATCAT
CMVGFP_27_7	CCCCGCCCATTGACGTCAATAATGACGTATGTTCCCTTTCATCATCAT
CMVGFP_27_8	GTGGAGTATTTACGGTAAACTGCCCACTTGGCAGTATTTCATCATCAT
CMVGFP_27_9	CCCCTATTGACGTCAATGACGGTAAATGGCCCGCCTTTTCATCATCAT
CMVGFP_27_10	GGCATTATGCCCAGTACATGACCTTATGGGACTTTCCTTTCATCATCAT
CMVGFP_27_11	TGGCAGTACATCTACGTATTAGTCATCGCTATTACCATGGTTTTCATCATCAT
CMVGFP_27_12	GATGCGGTTTTGGCAGTACATCAATGGGCGTGGATATTTCATCATCAT
CMVGFP_27_13	CGGTTTGACTCACGGGGATTTCCAAGTCTCCACCCCTTTCATCATCAT
CMVGFP_27_14	ATTGACGTCAATGGGAGTTTGTTTTGGCACCAAAATCATTTCATCATCAT
CMVGFP_27_15	ACGGGACTTTCCAAAATGTCGTAACAACTCCGCCCCTTTCATCATCAT
CMVGFP_27_16	CAAATGGGCGGTAGGCGTGTACGGTGGGAGGTCTATATTTTCATCATCAT
CMVGFP_27_17	AAGCAGAGCTCGTTTAGTGAACCGTCAGATCGCCTGTTTCATCATCAT
CMVGFP_27_18	CGATCCAGCCTCCCCTCGAAGCTGATCCTGAGAACTTTTCATCATCAT
CMVGFP_27_19	TCAGGGTGAGTCTATGGGACCCTTGATGTTTTCTTTCTTTCATCATCAT
CMVGFP_27_20	ATGGTTAAGTTCATGTCATAGGAAGGGGAGAAGTAACAGGGTTTCATCATCAT
CMVGFP_27_21	TCAGGGCAATAATGATACAATGTATCATGCCTCTTTGCACCTTTCATCATCAT
CMVGFP_27_22	TCATATTGCTAATAGCAGCTACAATCCAGCTACCATTCTGCTTTCATCATCAT
CMVGFP_27_23	TTTTATGGTTGGGATAAGGCTGGATTATTCTGAGTCCAAGCTTTCATCATCAT
CMVGFP_27_24	GGCCCTTTTGCTAATCATGTTCATACCTCTTATCTTCCTCCTTTCATCATCAT
CMVGFP_27_25	CTGGTCTGTGTGCTGGCCCATCACTTTGGCAAAGAATTTCATCATCAT
CMVGFP_27_26	GCTACCCCGACCACATGAAGCAGCACGACTTCTTCATTTCATCATCAT
CMVGFP_27_27	CGAAGGCTACGTCCAGGAGCGCACCATCTTCTTCAATTTCATCATCAT
CMVGFP_27_28	GTGAACCGCATCGAGCTGAAGGGCATCGACTTCAAGTTTCATCATCAT
CMVGFP_27_29	CGGCAACATCCTGGGGCACAAGCTGGAGTACAACTATTTCATCATCAT
CMVGFP_27_30	CAACAGCCACAACGTCTATATCATGGCCGACAAGCATTTCATCATCAT
CMVGFP_27_31	GAAGAACGGCATCAAGGTGAACTTCAAGATCCGCCATTTCATCATCAT
CMVGFP_27_32	CTGAGCAAAGACCCCAACGAGAAGCGCGATCACATGTTTCATCATCAT
CMVGFP_27_33	CCGGGATCACTCTCGGCATGGACGAGCTGTACAAGTATTTCATCATCAT
CMVGFP_27_34	AAGCGGCCGCTCTAGAGGATCCAAGCTTATCGATACTTTCATCATCAT
CMVGFP_27_35	CCAGATCTAATTCACCCCACCAGTGCAGGCTGCCTATTTCATCATCAT
CMVGFP_27_36	CTGGTGTGGCTAATGCCCTGGCCCACAAGTATCACTTTTCATCATCAT
CMVGFP_27_37	AAGCTCGCTTTCTTGCTGTCCAATTTCTATTAAAGGTTCCTTTTCATCATCAT
CMVGFP_27_38	GGGGATATTATGAAGGGCCTTGAGCATCTGGATTCTTTTCATCATCAT
CMVGFP_27_39	ATATCTTAAACTCCATGAAAGAAGGTGAGGCTGCAAACAGCTTTCATCATCAT
CMVGFP_27_40	TAATGCACATTGGCAACAGCCCCTGATGCCTATGCCTTTCATCATCAT
CMVGFP_27_41	TTCATCCCTCAGAAAAGGATTCAAGTAGAGGCTTGATTTGGTTTCATCATCAT
CMVGFP_27_42	TTTAGCTGTCCTCATGAATGTCTTTTCACTACCCATTTGCTTTTCATCATCAT
CMVGFP_27_43	TATCCTGCATCTCTCAGCCTTGACTCCACTCAGTTCTTTCATCATCAT
CMVGFP_27_44	TCTTGCTTAGAGATACCACCTTTCCCCTGAAGTGTTCCTTTCATCATCAT
CMVGFP_27_45	TTCCATGTTTTACGGCGAGATGGTTTCTCCTCGCCTTTTCATCATCAT
CMVGFP_27_46	GGCCACTCAGCCTTAGTTGTCTCTGTTGTCTTATAGAGGTTTCATCATCAT
CMVGFP_27_47	CCCCACTCACAGTGACCCGGAATCCCTCGACATCTATTTCATCATCAT

Table S2. SABER-FISH probe sequences for 5' and 3' halves of AAV8-CMV-GFP.

ID	*Sequence*
AAV-5'_28_1	CAGAGAGGGAGTGGCCAACTCCATCACTAGGGGTTCTTTCAACTTAAC
AAV-5'_28_2	AGTTAATGATTAACCCGCCATGCTACTTATCTACGTAGCCATTTCAACTTAAC
AAV-5'_28_3	AAAATTAGTCAGCCATGAGCTTGGCCCATTGCATACTTTCAACTTAAC
AAV-5'_28_4	TGTACATTTATATTGGCTCATGTCCAACATTACCGCCATGTTTTCAACTTAAC
AAV-5'_28_5	ATTACGGGGTCATTAGTTCATAGCCCATATATGGAGTTCCGTTTCAACTTAAC
AAV-5'_28_6	CGTTACATAACTTACGGTAAATGGCCCGCCTGGCTGTTTCAACTTAAC
AAV-5'_28_7	CCCCGCCCATTGACGTCAATAATGACGTATGTTCCCTTTCAACTTAAC
AAV-5'_28_8	GTGGAGTATTTACGGTAAACTGCCCACTTGGCAGTATTTCAACTTAAC
AAV-5'_28_9	CCCCTATTGACGTCAATGACGGTAAATGGCCCGCCTTTTCAACTTAAC
AAV-5'_28_10	GGCATTATGCCCAGTACATGACCTTATGGGACTTTCCTTTCAACTTAAC
AAV-5'_28_11	TGGCAGTACATCTACGTATTAGTCATCGCTATTACCATGGTTTTCAACTTAAC
AAV-5'_28_12	GATGCGGTTTTGGCAGTACATCAATGGGCGTGGATATTTCAACTTAAC
AAV-5'_28_13	CGGTTTGACTCACGGGGATTTCCAAGTCTCCACCCCTTTCAACTTAAC
AAV-5'_28_14	ATTGACGTCAATGGGAGTTTGTTTTGGCACCAAAATCATTTCAACTTAAC
AAV-5'_28_15	ACGGGACTTTCCAAAATGTCGTAACAACTCCGCCCCTTTCAACTTAAC
AAV-5'_28_16	CAAATGGGCGGTAGGCGTGTACGGTGGGAGGTCTATATTTTCAACTTAAC
AAV-5'_28_17	AAGCAGAGCTCGTTTAGTGAACCGTCAGATCGCCTGTTTCAACTTAAC
AAV-5'_28_18	CGATCCAGCCTCCCCTCGAAGCTGATCCTGAGAACTTTTCAACTTAAC
AAV-5'_28_19	TCAGGGTGAGTCTATGGGACCCTTGATGTTTTCTTTCTTTCAACTTAAC
AAV-5'_28_20	ATGGTTAAGTTCATGTCATAGGAAGGGGAGAAGTAACAGGGTTTCAACTTAAC
AAV-5'_28_21	TCAGGGCAATAATGATACAATGTATCATGCCTCTTTGCACCTTTCAACTTAAC
AAV-5'_28_22	TCATATTGCTAATAGCAGCTACAATCCAGCTACCATTCTGCTTTCAACTTAAC
AAV-5'_28_23	TTTTATGGTTGGGATAAGGCTGGATTATTCTGAGTCCAAGCTTTCAACTTAAC
AAV-5'_28_24	GGCCCTTTTGCTAATCATGTTCATACCTCTTATCTTCCTCCTTTCAACTTAAC
AAV-3'_27_25	CTGGTCTGTGTGCTGGCCCATCACTTTGGCAAAGAATTTCATCATCAT
AAV-3'_27_26	GCTACCCCGACCACATGAAGCAGCACGACTTCTTCATTTCATCATCAT
AAV-3'_27_27	CGAAGGCTACGTCCAGGAGCGCACCATCTTCTTCAATTTCATCATCAT
AAV-3'_27_28	GTGAACCGCATCGAGCTGAAGGGCATCGACTTCAAGTTTCATCATCAT
AAV-3'_27_29	CGGCAACATCCTGGGGCACAAGCTGGAGTACAACTATTTCATCATCAT
AAV-3'_27_30	CAACAGCCACAACGTCTATATCATGGCCGACAAGCATTTCATCATCAT
AAV-3'_27_31	GAAGAACGGCATCAAGGTGAACTTCAAGATCCGCCATTTCATCATCAT
AAV-3'_27_32	CTGAGCAAAGACCCCAACGAGAAGCGCGATCACATGTTTCATCATCAT
AAV-3'_27_33	CCGGGATCACTCTCGGCATGGACGAGCTGTACAAGTATTTCATCATCAT
AAV-3'_27_34	AAGCGGCCGCTCTAGAGGATCCAAGCTTATCGATACTTTCATCATCAT
AAV-3'_27_35	CCAGATCTAATTCACCCCACCAGTGCAGGCTGCCTATTTCATCATCAT
AAV-3'_27_36	CTGGTGTGGCTAATGCCCTGGCCCACAAGTATCACTTTTCATCATCAT
AAV-3'_27_37	AAGCTCGCTTTCTTGCTGTCCAATTTCTATTAAAGGTTCCTTTTCATCATCAT
AAV-3'_27_38	GGGGATATTATGAAGGGCCTTGAGCATCTGGATTCTTTTCATCATCAT
AAV-3'_27_39	ATATCTTAAACTCCATGAAAGAAGGTGAGGCTGCAAACAGCTTTCATCATCAT
AAV-3'_27_40	TAATGCACATTGGCAACAGCCCCTGATGCCTATGCCTTTCATCATCAT
AAV-3'_27_41	TTCATCCCTCAGAAAAGGATTCAAGTAGAGGCTTGATTTGGTTTCATCATCAT
AAV-3'_27_42	TTTAGCTGTCCTCATGAATGTCTTTTCACTACCCATTTGCTTTTCATCATCAT
AAV-3'_27_43	TATCCTGCATCTCTCAGCCTTGACTCCACTCAGTTCTTTCATCATCAT
AAV-3'_27_44	TCTTGCTTAGAGATACCACCTTTCCCCTGAAGTGTTCCTTTCATCATCAT
AAV-3'_27_45	TTCCATGTTTTACGGCGAGATGGTTTCTCCTCGCCTTTTCATCATCAT
AAV-3'_27_46	GGCCACTCAGCCTTAGTTGTCTCTGTTGTCTTATAGAGGTTTCATCATCAT
AAV-3'_27_47	CCCCACTCACAGTGACCCGGAATCCCTCGACATCTATTTCATCATCAT

Table S3. SABER-FISH probe sequences for AAV8-Best1-sCX3CL1.

ID	*Sequence*
Best1sCX3CL1_28_1	TCCTTTTCAGATAAGGGCACTGAGGCTGAGAGAGGATTTCAACTTAAC
Best1sCX3CL1_28_2	ACCAGAAACCAGGACTGTTGACTGCAGCCCGGTATTTTTCAACTTAAC
Best1sCX3CL1_28_3	CATTCTTTCCATAGCCCACAGGGCTGTCAAAGACCCTTTCAACTTAAC
Best1sCX3CL1_28_4	AGGGCCTAGTCAGAGGCTCCTCCTTCCTGGAGAGTTTTTCAACTTAAC
Best1sCX3CL1_28_5	AACTCTCTCTGCAAGGCCTCAGGGGTCAGAACACTGTTTCAACTTAAC
Best1sCX3CL1_28_6	GTGGAGCAGATCCTTTAGCCTCTGGATTTTAGGGCCTTTCAACTTAAC
Best1sCX3CL1_28_7	ATGGTAGAGGGGGTGTTGCCCTAAATTCCAGCCCTGTTTCAACTTAAC
Best1sCX3CL1_28_8	GTCTCAGCCCAACACCCTCCAAGAAGAAATTAGAGGTTTCAACTTAAC
Best1sCX3CL1_28_9	AGGCTGTGCTAGCCGTTGCTTCTGAGCAGATTACAATTTCAACTTAAC
Best1sCX3CL1_28_10	GAAGGGACTAAGACAAGGACTCCTTTGTGGAGGTCCTTTCAACTTAAC
Best1sCX3CL1_28_11	TAGTCGCCAGACCCAGATCCTGCAGAAGTTGGTCGTTTTCAACTTAAC
Best1sCX3CL1_28_12	GAGGCACTGGGCAGGTAAGTATCAAGGTTACAAGACTTTCAACTTAAC
Best1sCX3CL1_28_13	AGGTTTAAGGAGACCAATAGAAACTGGGCTTGTCGATTTCAACTTAAC
Best1sCX3CL1_28_14	GACAGAGAAGACTCTTGCGTTTCTGATAGGCACCTATTTCAACTTAAC
Best1sCX3CL1_28_15	TTGGTCTTACTGACATCCACTTTGCCTTTCTCTCCATTTCAACTTAAC
Best1sCX3CL1_28_16	TTCTTCCATTTGTGTACTCTGCTGCCGGGTCAGCACTTTCAACTTAAC
Best1sCX3CL1_28_17	CTCGGCATGACGAAATGCGAAATCATGTGCGACAAGTTTCAACTTAAC
Best1sCX3CL1_28_18	TCACGAATCCCAGTGGCTTTGCTCATCCGCTATCAGTTTCAACTTAAC
Best1sCX3CL1_28_19	ATTGTCCTGGAGACGACACAGCACAGACGCTTCTGTTTTCAACTTAAC
Best1sCX3CL1_28_20	AAGGAGAAATGGGTCCAAGACGCCATGAAGCATCTGTTTCAACTTAAC
Best1sCX3CL1_28_21	GATCACCAGGCTGCTGCCCTCACTAAAAATGGTGGCTTTCAACTTAAC
Best1sCX3CL1_28_22	AAGTTTGAGAAGCGGGTGGACAATGTGACACCTGGGTTTCAACTTAAC
Best1sCX3CL1_28_23	TGACAAAGCCTGAATCCGCCACATTGGAAGACCTTGTTTCAACTTAAC
Best1sCX3CL1_28_24	CTTTGGAACTGACTACTATTTCCCAGGAGGCCAGGGTTTCAACTTAAC
Best1sCX3CL1_28_25	AGCAGTGACCGGATCATCTCTCTCAACTTCCGAGGCTTTCAACTTAAC
Best1sCX3CL1_28_26	TTACGGCTAAGCCTCAGAGCATTGGAAGTTTTGAGGTTTCAACTTAAC
Best1sCX3CL1_28_27	CGTTTGGCCGAGTCCTGCTGTCTACCAATCTGGATCTTTCAACTTAAC
Best1sCX3CL1_28_28	TAGCTCCTGGGCTGAGGAAAAAGCTACTGAGTCCCCTTTCAACTTAAC
Best1sCX3CL1_28_29	CTCCACTACAGCCCCATCTCCTCAGGTGTCCACTACTTTCAACTTAAC
Best1sCX3CL1_28_30	TTCACCTTCAACCCCAGAGGAAAATGTTGGGTCCGATTTCAACTTAAC
Best1sCX3CL1_28_31	TCCAGGGACAGGACCTCAGTCCAGAGAAGTCTCTAGTTTCAACTTAAC
Best1sCX3CL1_28_32	ACCCAGTTCATACTGATAATTTCCAGGAGAGGGGGCTTTCAACTTAAC
Best1sCX3CL1_28_33	AGCCAGGCTCCTAAGATAGAGGAACCCATCCATGCCTTTCAACTTAAC
Best1sCX3CL1_28_34	ACTGCAGATCCCCAGAAACTGAGTGTGCTTATCACTTTTCAACTTAAC
Best1sCX3CL1_28_35	ACACCCAGGCAGCCACATGAAAGCTTGGATCCAATCTTTCAACTTAAC
Best1sCX3CL1_28_36	CGCTATGTGGATACGCTGCTTTAATGCCTTTGTATCATGCTTTCAACTTAAC
Best1sCX3CL1_28_37	AATCCTGGTTGCTGTCTCTTTATGAGGAGTTGTGGCTTTCAACTTAAC
Best1sCX3CL1_28_38	ACTGACAATTCCGTGGTGTTGTCGGGGAAATCATCGTTTCAACTTAAC
Best1sCX3CL1_28_39	CGCGTCTTCGAGATCTGCCTCGACTGTGCCTTCTAGTTTCAACTTAAC
Best1sCX3CL1_28_40	CCTGGAAGGTGCCACTCCCACTGTCCTTTCCTAATATTTCAACTTAAC
Best1sCX3CL1_28_41	TCGCATTGTCTGAGTAGGTGTCATTCTATTCTGGGGTTTCAACTTAAC
Best1sCX3CL1_28_42	AGGACAGCAAGGGGGAGGATTGGGAAGACAATAGCATTTCAACTTAAC
Best1sCX3CL1_28_43	ATGCTGGGGACTCGAGTTAAGGGCGAATTCCCGATATTTCAACTTAAC

Table S4. SABER-FISH probe sequences for AAV8-CMV-H2B-FusionRed.

ID	*Sequence*
CMVFusRed_28_1	CAGAGAGGGAGTGGCCAACTCCATCACTAGGGGTTCTTTCAACTTAAC
CMVFusRed_28_2	AGTTAATGATTAACCCGCCATGCTACTTATCTACGTAGCCATTTCAACTTAAC
CMVFusRed_28_3	AAAATTAGTCAGCCATGAGCTTGGCCCATTGCATACTTTCAACTTAAC
CMVFusRed_28_4	TGTACATTTATATTGGCTCATGTCCAACATTACCGCCATGTTTTCAACTTAAC
CMVFusRed_28_5	ATTACGGGGTCATTAGTTCATAGCCCATATATGGAGTTCCGTTTCAACTTAAC
CMVFusRed_28_6	CGTTACATAACTTACGGTAAATGGCCCGCCTGGCTGTTTCAACTTAAC
CMVFusRed_28_7	CCCCGCCCATTGACGTCAATAATGACGTATGTTCCCTTTCAACTTAAC
CMVFusRed_28_8	GTGGAGTATTTACGGTAAACTGCCCACTTGGCAGTATTTCAACTTAAC
CMVFusRed_28_9	CCCCTATTGACGTCAATGACGGTAAATGGCCCGCCTTTTCAACTTAAC
CMVFusRed_28_10	GGCATTATGCCCAGTACATGACCTTATGGGACTTTCCTTTCAACTTAAC
CMVFusRed_28_11	TGGCAGTACATCTACGTATTAGTCATCGCTATTACCATGGTTTTCAACTTAAC
CMVFusRed_28_12	GATGCGGTTTTGGCAGTACATCAATGGGCGTGGATATTTCAACTTAAC
CMVFusRed_28_13	CGGTTTGACTCACGGGGATTTCCAAGTCTCCACCCCTTTCAACTTAAC
CMVFusRed_28_14	ATTGACGTCAATGGGAGTTTGTTTTGGCACCAAAATCATTTCAACTTAAC
CMVFusRed_28_15	ACGGGACTTTCCAAAATGTCGTAACAACTCCGCCCCTTTCAACTTAAC
CMVFusRed_28_16	CAAATGGGCGGTAGGCGTGTACGGTGGGAGGTCTATATTTCAACTTAAC
CMVFusRed_28_17	AAGCAGAGCTCGTTTAGTGAACCGTCAGATCGCCTGTTTCAACTTAAC
CMVFusRed_28_18	CGATCCAGCCTCCCCTCGAAGCTGATCCTGAGAACTTTTCAACTTAAC
CMVFusRed_28_19	TCAGGGTGAGTCTATGGGACCCTTGATGTTTTCTTTCTTTCAACTTAAC
CMVFusRed_28_20	ATGGTTAAGTTCATGTCATAGGAAGGGGAGAAGTAACAGGGTTTCAACTTAAC
CMVFusRed_28_21	TCAGGGCAATAATGATACAATGTATCATGCCTCTTTGCACCTTTCAACTTAAC
CMVFusRed_28_22	TCATATTGCTAATAGCAGCTACAATCCAGCTACCATTCTGCTTTCAACTTAAC
CMVFusRed_28_23	TTTTATGGTTGGGATAAGGCTGGATTATTCTGAGTCCAAGCTTTCAACTTAAC
CMVFusRed_28_24	GGCCCTTTTGCTAATCATGTTCATACCTCTTATCTTCCTCCTTTCAACTTAAC
CMVFusRed_28_25	GAGCTCAAGCTTCGAATTCTGCAGTCGACGGTACCGTTTCAACTTAAC
CMVFusRed_28_26	CAAGCGCAGCCGCAAGGAGAGCTATTCCATCTATGTTTCAACTTAAC
CMVFusRed_28_27	GTACAAGGTTCTGAAGCAGGTCCACCCTGACACCGGTTTCAACTTAAC
CMVFusRed_28_28	CATTTCGTCCAAGGCCATGGGCATCATGAATTCGTTTTTCAACTTAAC
CMVFusRed_28_29	ATTACAACAAGCGCTCGACCATCACCTCCAGGGAGATTTCAACTTAAC
CMVFusRed_28_30	ATCACCAAGTACACCAGCGCTAAGGATCCACCGGTCTTTCAACTTAAC
CMVFusRed_28_31	GCCACCATGGTGAGCGAGCTGATTAAGGAGAACATGTTTCAACTTAAC
CMVFusRed_28_32	CCCATGAAGCTGTACATGGAGGGCACCGTGAACAACTTTCAACTTAAC
CMVFusRed_28_33	ACATCCTGGCTACCAGCTTCATGTACGGCAGCAGAATTTCAACTTAAC
CMVFusRed_28_34	ACTTCTTTAAGCAGTCCTTCCCTGAGGGCTTCACATTTTCAACTTAAC
CMVFusRed_28_35	TCATCTACAACGTCAAGGTTAGAGGGGTGAACTTCCTTTCAACTTAAC
CMVFusRed_28_36	CAGCCAACGGCCCTGTGATGCAGAAGAAAACACTCGTTTCAACTTAAC
CMVFusRed_28_37	GCCACCTGATCTGCAACCTTGAGACCACATACAGATTTCAACTTAAC
CMVFusRed_28_38	GGCGTCTACAACGTGGACCACAGACTGGAAAGAATCTTTCAACTTAAC
CMVFusRed_28_39	CCAGATCTAATTCACCCCACCAGTGCAGGCTGCCTATTTCAACTTAAC
CMVFusRed_28_40	CTGGTGTGGCTAATGCCCTGGCCCACAAGTATCACTTTTCAACTTAAC
CMVFusRed_28_41	AAGCTCGCTTTCTTGCTGTCCAATTTCTATTAAAGGTTCCTTTTCAACTTAAC
CMVFusRed_28_42	GGGGATATTATGAAGGGCCTTGAGCATCTGGATTCTTTTCAACTTAAC
CMVFusRed_28_43	ATATCTTAAACTCCATGAAAGAAGGTGAGGCTGCAAACAGCTTTCAACTTAAC
CMVFusRed_28_44	TAATGCACATTGGCAACAGCCCCTGATGCCTATGCCTTTCAACTTAAC
CMVFusRed_28_45	TTCATCCCTCAGAAAAGGATTCAAGTAGAGGCTTGATTTGGTTTCAACTTAAC
CMVFusRed_28_46	TTTAGCTGTCCTCATGAATGTCTTTTCACTACCCATTTGCTTTTCAACTTAAC
CMVFusRed_28_47	TATCCTGCATCTCTCAGCCTTGACTCCACTCAGTTCTTTCAACTTAAC
CMVFusRed_28_48	TCTTGCTTAGAGATACCACCTTTCCCCTGAAGTGTTCCTTTCAACTTAAC
CMVFusRed_28_49	TTCCATGTTTTACGGCGAGATGGTTTCTCCTCGCCTTTTCAACTTAAC
CMVFusRed_28_50	GGCCACTCAGCCTTAGTTGTCTCTGTTGTCTTATAGAGGTTTCAACTTAAC
CMVFusRed_28_51	CCCCACTCACAGTGACCCGGAATCCCTCGACATCTATTTCAACTTAAC

Table S5. Catalytic hairpin and fluorescent oligonucleotide sequences.

ID	*Sequence*
Hairpin_27	ACATCATCATGGGCCTTTTGGCCCATGATGATGTATGATGATGTTTTTTT
Hairpin_28	ACAACTTAACGGGCCTTTTGGCCCGTTAAGTTGTGTTAAGTTGTTTTTTT
Fluor_27	/5ATTO550N/TTATGATGATGTATGATGATGT
Fluor_28	/5ATTO633N/TTGTTAAGTTGTGTTAAGTTGT

Table S6. qPCR Primers.

Target	*5' Sequence*	*3' Sequence*
FusionRed (DNA)	CTCCACCGAGACGATGTACC	TGAGGTTCGTAGCGGGTTTC
Gapdh (DNA)	GGTTGTCTCCTGCGACTTCA	CAGGTTTCCCATCCCCACAT
Gapdh (RNA)	AGGTCGGTGTGAACGGATTTG	TGTAGACCATGTAGTTGAGGTCA
Opn1mw (RNA)	ATGGCCCAAAGGCTTACAGG	CCACAAGAATCATCCAGGTGC
Rho (RNA)	CCCTTCTCCAACGTCACAGG	TGAGGAAGTTGATGGGGAAGC
Tmem119 (RNA)	CCTACTCTGTGTCACTCCCG	CACGTACTGCCGGAAGAAATC

SUPPLEMENTAL REFERENCES

1. Wang, S.K., Xue, Y., Rana, P., Hong, C.M. and Cepko, C.L. (2019). Soluble CX3CL1 gene therapy improves cone survival and function in mouse models of retinitis pigmentosa. *Proc. Natl. Acad. Sci. U. S. A.* 116: 10140–10149.
2. Mosaliganti, K.R., Noche, R.R., Xiong, F., Swinburne, I.A. and Megason, S.G. (2012). ACME: Automated Cell Morphology Extractor for Comprehensive Reconstruction of Cell Membranes. *PLoS Comput. Biol.* 8.
3. Kishi, J.Y., Lapan, S.W., Beliveau, B.J., West, E.R., Zhu, A., Sasaki, H.M., Saka, S.K., Wang, Y., Cepko, C.L. and Yin, P. (2019). SABER amplifies FISH: enhanced multiplexed imaging of RNA and DNA in cells and tissues. *Nat. Methods* 16: 533–544.
4. Bennett, M.L., Bennett, F.C., Liddelow, S.A., Ajami, B., Zamanian, J.L., Fernhoff, N.B., Mulinyawe, S.B., Bohlen, C.J., Adil, A., Tucker, A., *et al.* (2016). New tools for studying microglia in the mouse and human CNS. *Proc. Natl. Acad. Sci. U. S. A.* 113: E1738–E1746.
